# Sequential gemcitabine–docetaxel in BCG-naïve and BCG-failure non–muscle-invasive bladder cancer: a systematic review and meta-analysis

**DOI:** 10.3389/fonc.2026.1859461

**Published:** 2026-07-09

**Authors:** Bandar Alhubaishy, Ibrahim Beshawri, Inam Abulreish, Omar Alnajar, Hamad Radhi, Abduljawad Saleh, Saher Alwafi, Abdulghafour Halawani

**Affiliations:** 1 Department of Urology, King Abdulaziz University, Jeddah, Saudi Arabia; 2 King Abdulaziz University Hospital, Jeddah, Saudi Arabia; 3 King Saud bin Abdulaziz University for Health Science، Collage of Medicine, Jeddah, Saudi Arabia; 4 Umm Al-Qura University, College of Medicine, Makkah, Saudi Arabia; 5 King Abdulaziz University, Collage of Medicine - Rabigh Branch, Jeddah, Saudi Arabia

**Keywords:** BCG (Bacille Calmette-Guerin), BCG-naïve, BCG-unresponsive, docetaxel, gemcitabine, intravesical therapy, non-muscle invasive bladder cancer, BCG-failure

## Abstract

**Background:**

Non–muscle-invasive bladder cancer (NMIBC) is associated with a high recurrence rate and the potential to progress, especially among high-risk patients. Limitations of Bacillus Calmette–Guérin (BCG), including treatment failure, high recurrence rates, adverse events, and global shortages, create an unmet need for alternative agents. Objective: To assess the efficacy and safety of intravesical gemcitabine and docetaxel (GEM/DOCE) in the treatment of naïve and BCG-failure high-risk and very high-risk NMIBC.

**Methods:**

A systematic review and meta-analysis was prospectively registered (PROSPERO: CRD420251050582) and conducted in accordance with PRISMA 2020 guidelines. Databases were searched from January 2014 to March 2025. Eligible studies enrolled adult patients with NMIBC treated with intravesical sequential GEM/DOCE. Data were synthesized using random-effects and common-effects meta-analysis models for oncological and safety outcomes, incorporating subgroup analyses by prior BCG status.

**Results:**

Nine studies comprising 883 patients were included. At 12 months, the pooled recurrence-free survival (RFS) was 73.75% and high-grade recurrence-free survival (HG-RFS) was 75.78%. BCG-naïve patients demonstrated significantly superior 12-month RFS (82.50% vs. 60.00%, p < 0.001) and HG-RFS (84.09% vs. 63.79%, p < 0.001) compared to BCG-failure cohorts. Progression-free survival (95.57%), cancer-specific survival (99.18%), overall survival (97.32%), and cystectomy-free survival (94.41%) remained high at 12 months. GEM/DOCE was generally well tolerated. The pooled proportion of patients experiencing treatment-related adverse events was 52.59%, with an overall treatment intolerance rate of only 3.55%.

**Conclusion:**

Intravesical GEM/DOCE represents an effective and well-tolerated intravesical therapeutic option in patients with high- and very high-risk NMIBC, demonstrating favorable recurrence outcomes with preserved survival. The regimen appears particularly effective in BCG-naïve disease, while also providing clinically meaningful disease control in BCG-failure populations. However, the available evidence is limited by heterogeneity across studies and the predominance of retrospective designs. Further prospective, adequately powered studies are required to better define its role and optimize treatment protocols.

**Systematic Review Registration:**

https://www.crd.york.ac.uk/PROSPERO/view/CRD420251050582, identifier CRD420251050582.

## 1 Introduction

Bladder cancer is the world’s 9th most frequently diagnosed malignancy, with 614,000 new cases and 220,000 related deaths estimated in 2022 (1). It predominantly affects men, with a male-to-female ratio of near 3:1, and its incidence increases with advancing age (1). Approximately 75% of all bladder cancer cases are non-muscle invasive (NMIBC), which carries a more favorable prognosis. Nevertheless, it is characterized by a high recurrence rate and the potential to progress into muscle-invasive bladder cancer (MIBC), especially among intermediate- and high-risk patients, despite initial local treatment (2).

Management of NMIBC usually involves transurethral resection of bladder tumor (TURBT), with subsequent instillation of intravesical therapy to reduce recurrence rates (2). The choice of intravesical therapy is guided by tumor risk stratification, prior Bacillus Calmette–Guérin (BCG) response, and patient and treatment factors, as well as histological features. BCG is the most widely used immunotherapy in intermediate- and high-risk NMIBC; however, treatment failure, high recurrence rates, adverse events, and global BCG shortage are all challenges that create an unmet need for alternative agents (3).

In view of these challenges, single immediate intravesical instillation using chemotherapeutic agents like mitomycin C (MMC), epirubicin, pirarubicin, or gemcitabine has been shown to reduce recurrence rates, with these agents, along with valrubicin and gemcitabine-docetaxel (GEM/DOCE), serving as commonly used alternatives in BCG-naïve patients. However, this approach should be avoided in patients with high risk of recurrence due to its lack of efficacy in this subgroup. For patients where BCG treatment has proved ineffective, bladder-sparing alternatives consist of nadofaragene firadenovec, sequential gemcitabine-docetaxel, systemic pembrolizumab, and valrubicin. Clinical trials have emphasized the efficacy of these options: intravesical nadofaragene firadenovec reached a 53.4% complete response rate in BCG-unresponsive carcinoma *in situ* (CIS), while systemic pembrolizumab achieved a 40% complete response rate (4–6).

Nonetheless, the need for an accessible, effective, and tolerable intravesical alternative that can address both BCG-naïve and BCG-failure disease remains, underscoring the need for rigorous studies evaluating such regimens to optimize protocols and improve patient outcomes.

One promising alternative is intravesical GEM/DOCE, which has recently demonstrated potential efficacy and safety in both BCG-naïve and BCG-failure NMIBC. A recent systematic review and meta-analysis found encouraging results, with 1-year and 2-year recurrence-free survival rates exceeding 80% in BCG-naïve patients. In the BCG-unresponsive cohort, 6-month, 1-year, and 2-year high-grade recurrence-free survival rates were 80%, 66%, and 51%, respectively, alongside a favorable tolerability profile (7). While these findings indicate the potential role of this therapy as an effective and safe alternative, the efficacy and safety of intravesical GEM/DOCE across BCG-naïve and BCG-failure high-risk NMIBC have not been comprehensively synthesized. This underscores the need for a rigorous and standardized systematic review of this regimen.

Given the global burden of NMIBC and the limitations of current standards, this systematic review aims to assess the efficacy and safety of intravesical GEM/DOCE in the treatment of naïve and BCG-failure high-risk and very high-risk NMIBC.

## 2 Methodology

### 2.1 Protocol and registration

This systematic review was prospectively registered in the International Prospective Register of Systematic Reviews (PROSPERO; ID: CRD420251050582) before study initiation and was conducted in accordance with the PRISMA 2020 reporting guidelines (8).

### 2.2 Search strategy

An extensive literature search was conducted in March 2025 in PubMed/MEDLINE, Web of Science, the Cochrane Library, and Google Scholar to identify studies published from 1 January 2014 to 31 March 2025 assessing intravesical sequential gemcitabine and docetaxel (GEM/DOCE) for NMIBC. The PubMed search used a combination of keywords as follows: (“gemcitabine” OR “docetaxel”) AND (“intravesical” OR “instillation”) AND (“bladder cancer” OR “urothelial cancer” OR “non–muscle-invasive bladder cancer” OR “NMIBC”). The same search string was applied in Web of Science and the Cochrane Library; Google Scholar was used to identify additional relevant reports. The search was restricted to human studies published in English, with no restrictions on study design beyond the eligibility criteria described below.

### 2.3 Study selectionc

**Figure 1:**
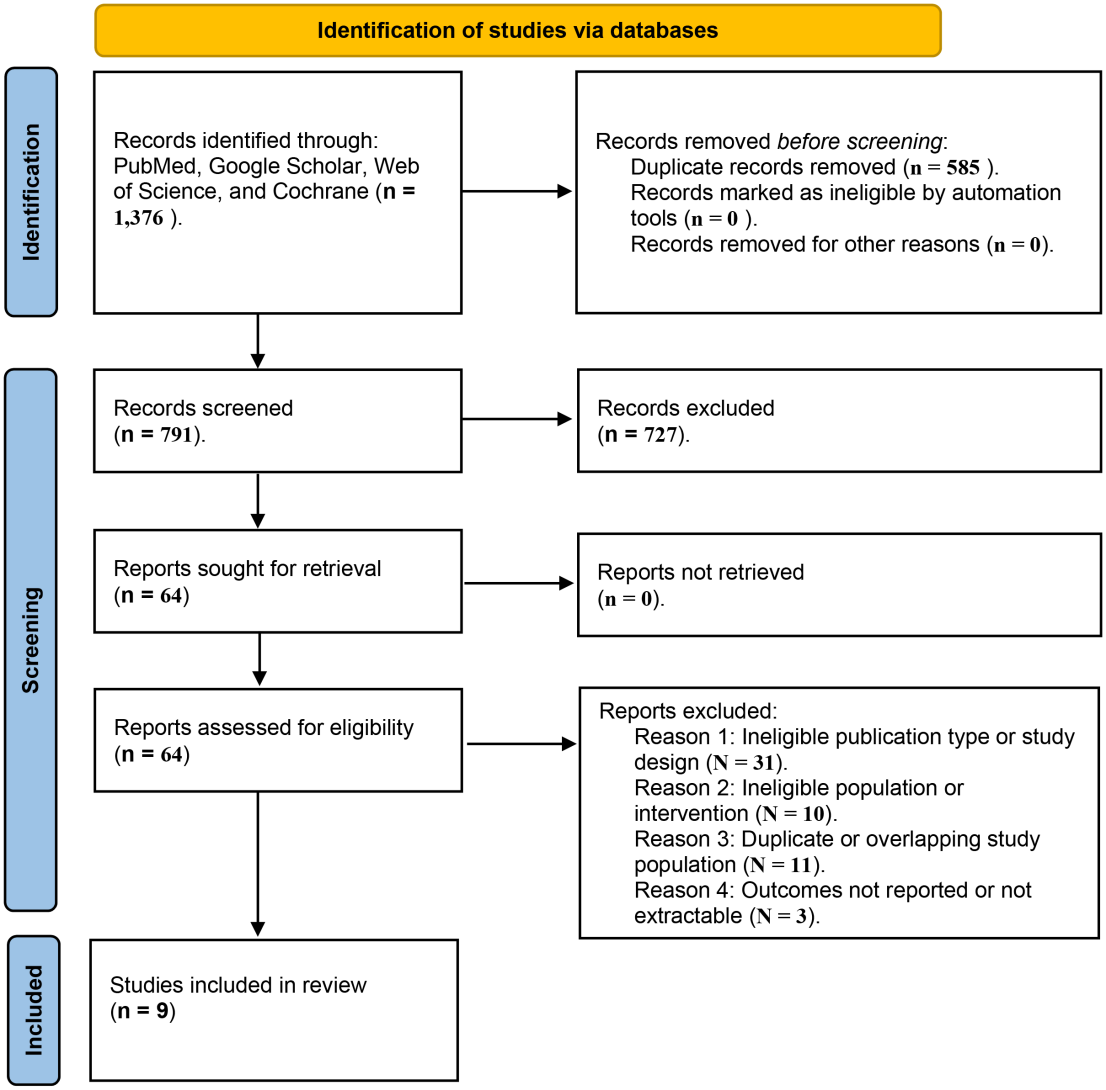
PRISMA 2020 flow diagram for new systematic reviews which included searches of databases and registers only.

All records identified through the database searches were uploaded into Rayyan, and duplicate entries were deleted. Titles and abstracts were then screened independently by two reviewers to omit clearly irrelevant reports, after which the full texts of potentially eligible articles were retrieved and evaluated in detail against the predefined inclusion and exclusion criteria. Any disagreements at either stage were resolved through discussion and, when required, consultation with a third reviewer. The study selection process, including the number of records identified, screened, excluded (with main reasons for exclusion at the full-text stage), and included in the qualitative synthesis, is summarized in the PRISMA flow diagram (**Figure 1**).

### 2.4 Inclusion/exclusion criteria

Studies were eligible for inclusion if they enrolled adult patients with NMIBC treated with intravesical sequential GEM/DOCE and reported oncologic and/or safety outcomes. Randomized controlled trials and prospective or retrospective cohort studies published as full-text original articles were included, irrespective of comparator, provided that GEM/DOCE was administered as an intravesical regimen. Review articles, editorials, letters, conference abstracts, case reports, non-human (animal or *in vitro*) studies, and articles not published in English were omitted.

### 2.5 Data extraction

Two investigators separately extracted data from each included study utilizing a uniform form. Extracted information included study characteristics (first author, year of publication, country, study design, study period); patient and tumor characteristics (sample size, sample size in the GEM/DOCE group, comparator regimen where applicable, mean or median age, sex distribution, follow-up duration, NMIBC risk category, BCG treatment history, and BCG-failure subcategory classification, encompassing BCG-unresponsive, BCG-refractory, BCG-relapsing, and BCG-intolerant disease, tumor stage and grade, presence of carcinoma *in situ*, and histological subtype); treatment details (GEM/DOCE dosing, dwell time, number of instillations, induction schedule, maintenance schedule, and the number of patients completing induction and receiving maintenance); and outcomes. Oncologic outcomes consisted of recurrence-free survival, high-grade recurrence-free survival, progression-free survival, cancer-specific survival, overall survival, cystectomy-free survival, and rates and indications of radical cystectomy; safety outcomes included the incidence and type of treatment-related adverse events, treatment intolerance, and treatment schedule modifications or discontinuation. Any discrepancies in extracted data were resolved by consensus among the reviewers.

### 2.6 Quality assessment and risk of bias

The methodological quality and risk of bias of the included studies were evaluated independently by two reviewers using the National Heart, Lung, and Blood Institute (NHLBI) Study Quality Assessment Tools, selecting the tool appropriate to each study design. Each item within the tool was rated as “Yes,” “No,” “Cannot determine,” “Not applicable,” or “Not reported,” and an overall quality rating of “good,” “fair,” or “poor” was assigned to each study (9). Conflicts among reviewers were resolved through discussion and, when required, consultation with a third reviewer. The results of the quality assessment are outlined narratively in the Results section and in **Supplementary Table 1**.

### 2.7 Statistical analysis

All meta-analyses were performed using RStudio (RStudio 2024.09.1 Build 394, Boston, MA, USA) and R version 4.5.1 with the metafor package. Proportional analyses were performed for six oncological outcomes (recurrence-free survival, high-grade recurrence-free survival, progression-free survival, cancer-specific survival, overall survival, and cystectomy-free survival) at multiple time points (6, 12, 24, and 36 months). Heterogeneity was quantified using I² and τ² statistics with maximum likelihood estimation. For outcomes with substantial heterogeneity (I² > 50%), a random-effects model was applied; otherwise, a common-effects model was used. The logit transformation was applied to proportions for meta-analysis computation. However, for boundary proportions (0% or 100%), the Freeman-Tukey double arcsine transformation was applied to stabilize variance and provide meaningful confidence intervals. Safety analyses were conducted for adverse event outcomes reported in four or more studies to ensure adequate power for subgroup comparisons. Subgroup analyses by BCG status (naïve versus failure) were performed for all outcomes, with between-group differences assessed using Q-tests. All proportional estimates are presented with 95% confidence intervals.

## 3 Result

### 3.1 Study selection

A sum of 1,376 records were identified through database searches in PubMed, Google Scholar, Web of Science, and the Cochrane Library. After removal of 585 duplicates, 791 unique records remained and were screened by title and abstract, leading to the exclusion of 727 records that did not meet the inclusion criteria. The remaining 64 reports were obtained in full and assessed for eligibility, after which 55 were excluded due to ineligible publication type or study design, ineligible population or intervention, duplicate or overlapping study population, and outcomes not reported or not extractable. Ultimately, 9 studies met all eligibility criteria and were included in the qualitative synthesis (**Figure 1**).

### 3.2 Study characteristics

#### 3.2.1 Study design and setting

The effectiveness and safety of intravesical GEM/DOCE combination therapy in patients with NMIBC were assessed in nine studies published between 2020 and 2025. Most studies were conducted in the United States, with additional data from one multicenter European cohort, including centers from France, Italy, Spain, Poland, Austria, and Germany, and one study from Croatia (10, 11). The included studies comprised a prospective observational study (12), a prospective clinical trial (11), and seven retrospective cohort studies (10, 13–18). Across all studies, the total pooled sample size was 883 participants, with individual study populations ranging from 25 to 276 patients (**Table 1**).

**Table 1:** Baseline study and patient characteristics.

Author, year	Country	Study design	Gem-doce population	Age	Gender distribution	Follow up (months)	NMIBC category	Tumor stage before treatment initiation	Risk group	Risk of bias judgment
McElree, 2023 (13)	USA	Retrospective	138	74 (IQR: 67 - 82)	Male: 116 (84.1%) Female: 22 (15.9%)	23 (IQR: 12-33)	BCG Naive	CIS only = 17 (12.3%) T1 = 56 (40.6%) T1 + CIS = 20 (14.5%) Ta = 26 (18.8%) Ta + CIS = 19 (13.8%)	High Risk	Good
Patel, 2024 (12)	USA	Prospective	25	68.9 (IQR: 59.9-73.8)	Male: 21 (84%) Female: 4 (16%)	19.6 (IQR: NR)	BCG Naive	CIS only = 1 (4%) T1-HG = 13 (52%) T1-HG + CIS = 3 (12%) Ta-HG = 7 (28%) Ta-HG + CIS = 1 (4%)	High Risk	Good
Bakula, 2024 (11)	Croatia	Prospective	52	68.2 (Range: 44.7–93)	Male: 44 (84.6%) Female: 8 (15.4%)	NR	BCG Naive	CIS only = 10 (19.2%) T1-HG = 25 (48.1%) T1-HG + CIS = 17 (32.7%)	High Risk	Good
Refugia, 2024 (14)	USA	Retrospective	53	73 (IQR 66-82)	Male: 36 (68%) Female: 17 (32%)	18 (IQR: NR)	BCG Naive	CIS only = 1 (2%) T1 = 14 (26%) T1 + CIS = 6 (11%) Ta = 29 (55%) Ta + CIS = 3 (6%)	High Risk	Good
Abou Chakra, 2025 (15)	USA	Retrospective	65	78 (IQR 72-82)	Male: 56 (86.2%) Female: 9 (13.8%)	23 (IQR 15−36)	BCG Naive	T1 = 30 (46.2%) T1 + CIS = 21 (32.3%) Ta = 3 (4.6%) Ta + CIS = 11 (16.9%)	Very High Risk	Good
Steinberg, 2020 (16)	USA, Canada	Retrospective	276	73 (Range: 43 - 94)	Male: 224 (81.2%) Female: 52 (18.8%)	22.9 (Range: 1.9 - 108)	BCG Failure	Any CIS = 173 (62.6%) Ta/T1-HG = 72 (26%) Ta/T1-LG = 31 (11.2%)	High Risk	Poor
Yim, 2023 (17)	USA	Retrospective	102	72 (IQR: 66-77)	Male: 73 (71.5%) Female: 29 (28.4%)	18 (IQR: 11-26)	BCG Failure	CIS only = 22 (21.6%) T1-HG = 24 (23.5%) Ta-LG = 6 (5.9%) Ta-HG = 50 (49.0%)	High Risk	Fair
Chevuru, 2023 (18)	USA	Retrospective	97	73 (IQR: 67-79)	Male: 74 (76%) Female: 23 (24%)	49 (IQR: NR)	BCG Failure	CIS only = 45 (46%) T1-HG = 12 (12%) T1-HG + CIS = 10 (10%) Ta-HG = 16 (16%) Ta-HG + CIS = 14 (14%)	High Risk	Good
Scilipoti, 2025 (10)	France, Italy, Spain, Poland, Austria, Germany	Retrospective	75	71 (IQR: 65–75)	Male: 59 (79%) Female: 16 (21%)	9 (IQR: 5-14)	BCG Failure	CIS only = 17 (23%) T1-HG = 17 (23%) T1-HG + CIS = 11 (15%) Ta-HG = 25 (33%) Ta-HG + CIS = 5 (6.7%)	High Risk, and Very High Risk	Poor

IQR, Interquartile Range; NR, Not Reported; Gem/Doce, Gemcitabine/Docetaxel BCG, Bacillus Calmette–Guérin; NMIBC, Non-Muscle Invasive Bladder Cancer; CIS, Carcinoma In Situ; HG, High Grade; LG, Low Grade.

#### 3.2.2 Demographics

Across the included studies, the median or mean age of participants varied from 68 to 78 years, demonstrating an elderly population typical of NMIBC. All cohorts demonstrated a clear male predominance, with men comprising 68% to 86% of study populations. Follow-up duration varied from approximately 9 to 49 months, although most studies reported at least 12 months of follow-up (**Table 1**).

#### 3.2.3 Clinical and tumor characteristics

Among the included studies, most patients were classified as having high-risk NMIBC, while two studies, Abou Chakra et al. (15) and Scilipoti et al. (10), also included patients with very–high-risk criteria. Five studies evaluated BCG-naïve disease (11–15), whereas four focused on BCG-failure cohorts (10, 16–18), reflecting a mixture of treatment-naïve and previously treated populations. Overall, the included cohorts predominantly consisted of patients with aggressive, high-grade NMIBC, frequently characterized by Ta-High Grade or T1-High Grade disease and substantial Carcinoma *in situ* involvement (**Table 1**).

#### 3.2.4 Risk of bias assessment

Overall, nine studies were included in the qualitative synthesis (10–18). Based on the NHLBI quality appraisal, six studies were rated as having good methodological quality (11–15, 18), one was rated as fair (17), and two were judged to have poor quality (10, 16). The lower-quality ratings were primarily due to inconsistencies in population selection, particularly the nonuniform application of prespecified eligibility criteria, as well as insufficient clarity and consistency in reporting the GEM/DOCE exposure measures. Nonetheless, all included studies fulfilled the primary objective of evaluating the safety and efficacy of intravesical GEM/DOCE, thereby meeting the aim of this review, as shown in **Supplementary Table 1**.

### 3.3 Treatment protocol characteristics

#### 3.3.1 Gemcitabine and docetaxel dose

GEM/DOCE was administered as sequential intravesical therapy in all included studies. Gemcitabine was instilled first at a dose of 1 g diluted in 50 mL of sterile water or normal saline, with a dwell time of 60–90 minutes. Docetaxel was subsequently instilled at a dose of 37.5 mg diluted in 50 mL of normal saline, with a dwell time of 60–120 minutes. A few protocol changes were reported: Steinberg et al. included hyperthermic instillation at 42 °C for both drugs in selected centers (University of Arizona) (16), and Scilipoti et al. utilized either 37.5 mg or 40 mg of docetaxel, depending on institutional policy (10) (**Table 2**).

**Table 2:** Intravesical gemcitabine–docetaxel treatment protocols across included studies.

Author, year	Treatment regimen	Gemcitabine dose	Docetaxel dose	Induction protocol	Maintenance protocol	Number of patients who received maintenance
McElree, 2023	Gemcitabine and Docetaxel	1 g (in 50 ml of sterile water or normal saline for 90 minutes)	37.5 mg (dissolved in 50 ml normal saline for 90 - 120 minutes)	Once a week for 6 weeks	Monthly for up to 24 months.	112 (81.2%)
Patel, 2024 (12)	Gemcitabine and Docetaxel	1 g (in 50 ml of sterile water for 60 minutes)	37.5 mg (dissolved in 50 ml normal saline for 60 minutes)	Once a week for 6 weeks	Monthly for up to 24 months.	25 (100%)
Bakula, 2024 (11)	Gemcitabine and Docetaxel	1 g (in 50 ml of normal saline for 90 minutes)	37.5 mg (in 50 ml normal saline for 90 - 120 minutes)	Once a week for 6 weeks	Monthly for up to 12 or 24 months.	NR
Refugia, 2024 (14)	Gemcitabine and Docetaxel	1 gm of gemcitabine (in 50 ml sterile water for 60 minutes)	37.5 mg (dissolved in 50 ml sterile water for 60 minutes)	Once a week for 6 weeks	Monthly for up to 24 months.	NR
Abou Chakra, 2025 (15)	Gemcitabine and Docetaxel	1 g (in 50 ml of normal saline for 60-90 minutes)	37.5 mg (dissolved in 50 cc normal saline for 60-90 minutes)	Once a week for 6 weeks	Monthly for up to 24 months.	NR
Steinberg, 2020	Gemcitabine and Docetaxel	1 g (in 50 ml of sterile water or normal saline for 60-90 minutes), the University of Ari zona used concomitant hyperthermia with instillation by warming the agents to 42C and using warm water in the catheter balloon.	37.5 mg (dissolved in 50 ml normal saline for 60 - 120 minutes), the University of Ari zona used concomitant hyperthermia with instillation by warming the agents to 42C and using warm water in the catheter balloon	Once a week for 6 weeks	Monthly for up to 24 months. except for Beth Israel and University of Arizona, which performed maintenance in accordance with a SWOG schedule	170 (61.6%)
Yim, 2023 (17)	Gemcitabine and Docetaxel	1 g (in 50 ml of sterile water or normal saline for 90 minutes)	37.5 mg (dissolved in 50 ml normal saline for 90 - 120 minutes)	Once a week for 6 weeks	Monthly for up to 24 months.	68 (66.7%)
Chevuru, 2023 (18)	Gemcitabine and Docetaxel	1 g (in 50 ml of sterile water or normal saline for 90 minutes)	37.5 mg (dissolved in 50 ml normal saline for 90 - 120 minutes)	Once a week for 6 weeks	Monthly for up to 24 months.	NR
Scilipoti, 2025 (10)	Gemcitabine and Docetaxel	1 g (in 50 ml of normal saline for 60-90 minutes)	37.5 mg (dissolved in 50 ml normal saline for 60-120 minutes) or 40 mg (dissolved in 40 ml normal saline for 60-120 minutes)	Once a week for 6 weeks	Monthly for up to 10 or 24 months.	51 (68%)

NR, not reported; SWOG, Southwest Oncology Group.

#### 3.3.2 Induction and maintenance protocol

The induction regimen was largely uniform across the included studies, with nearly all cohorts receiving weekly intravesical GEM/DOCE instillations for six consecutive weeks to ensure consistent initial exposure. When applied, maintenance therapy was typically delivered on a monthly schedule for up to 24 months. Two studies reported variations in maintenance duration: Scilipoti et al. described a range of 10–24 months (10), while Bakula et al. reported maintenance courses ranging from 12 to 24 months (11). Steinberg et al. predominantly followed a standard 24-month monthly schedule across participating centers, except at Beth Israel and the University of Arizona, where the Southwest Oncology Group (SWOG) maintenance protocol was used (16). The proportion of patients who proceeded to maintenance therapy varied across studies, ranging from 61% to 100%, reflecting differences in reporting completeness and treatment adherence (**Table 2**).

### 3.4 Oncological outcomes

#### 3.4.1 Recurrence-free survival

**Figure 2:**
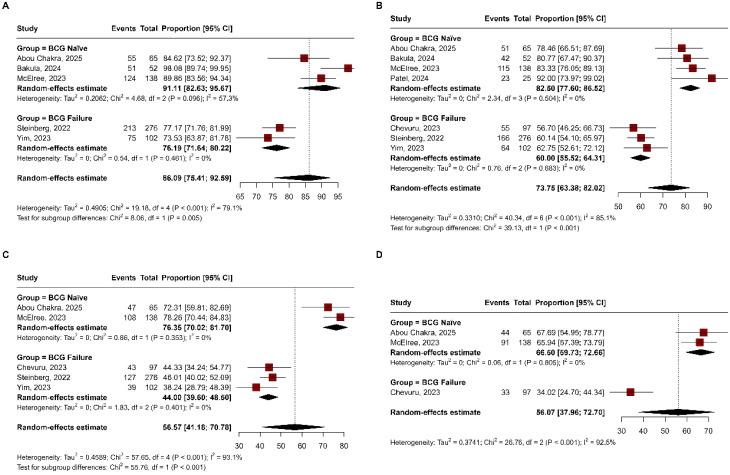
Forest plots of recurrence-free survival (RFS) at 6 months **(A)**, 12 months **(B)**, 24 months **(C)**, and 36 months **(D)**.

At 6 months, the pooled recurrence-free survival (RFS) was 86.09% (95% CI 75.41 92.59%), with moderate heterogeneity (I² = 79.1%, τ² = 0.4905). At 12 months, pooled RFS was 73.75% (95% CI 63.38–82.02%), with high heterogeneity (I² = 85.1%, τ² = 0.3310). At 24 months, pooled RFS declined to 56.57% (95% CI 41.18–70.78%), with very high heterogeneity (I² = 93.1%, τ² = 0.4589). At 36 months, pooled RFS was 56.07% (95% CI 37.96–72.70%), with very high heterogeneity (I² = 92.5%, τ² = 0.3741). BCG-naïve patients demonstrated superior RFS at 6 months (91.11%, 95% CI 82.63 95.67%) compared to BCG-failure patients (76.19%, 95% CI 71.64–80.22%), with significant subgroup difference (p = 0.005). At 12 months, BCG-naïve RFS was 82.50% (95% CI 77.60–86.52%) versus 60.00% (95% CI 55.52–64.31%) in BCG failure patients (p < 0.001). At 24 months, BCG-naïve RFS was 76.35% (95% CI 70.02–81.70%) compared to 44.00% (95% CI 39.60–48.50%) in BCG-failure patients (p < 0.001). At 36 months, BCG-naïve RFS was 66.50% (95% CI 59.73–72.66%) versus 34.02% (95% CI 24.70–44.34%) in BCG-failure patients (**Figure 2**, **Table 3**).

**Table 3:** Oncologic outcomes following intravesical gemcitabine–docetaxel therapy.

Author, year	RFS	HG RFS	PFS	CSS	OS	CFS	No. cystectomy	Reason of cystectomy
McElree, 2023	6 months: 90% (CI: 84%-94%) 12 months: 83% (CI: 75%-89%) 24 months: 78% (CI: 69%-85%) 36 months: 66% (CI: 53%-77%)	6 months: 92% (CI: 86%-95%) 12 months: 85% (CI: 78%-91%) 24 months: 81% (CI: 72%-87%) 36 months: 71% (CI: 58%-81%)	6 months: 99% (CI: 95%-100%) 12 months: 98% (CI: 93%-100%) 24 months: 97% (CI: 92%-99%) 36 months: 97% (CI: 92%-99%)	6 months: 100% (CI: 100%-100%) 12 months: 100% (CI: 100%-100%) 24 months: 100% (CI: 100%-100%) 36 months: 100% (CI: 100%-100%)	6 months: 98% (CI: 93%-99%) 12 months: 98% (CI: 93%-99%) 24 months: 89% (CI: 81%-94%) 36 months: 85% (CI: 73%-91%)	6 months: 99% (CI: 95%-100%) 12 months: 98% (CI: 93%-100%) 24 months: 98% (CI: 93%-100%) 36 months: 98% (CI: 93%-100%)	2 (1.4%)	Patients underwent cystectomy for end-stage bladder cancer symptoms
Patel, 2024 (12)	12 months: 92% (CI:90%-94%)	NR	NR	NR	NR	NR	NR	NR
Bakula, 2024 (11)	6 months: 98.1% 9 months: 94.2% 12 months: 80.8%	6 months: 98.1% 9 months: 94.2% 12 months: 84.6%	6 months: 100% 9 months: 98.1% 12 months: 92.3%	12 months: 100%	12 months: 98.1%	NR	1 (1.9%)	Radical cystectomy for T2 progression
Refugia, 2024 (14)	NR	6 months: 89% (CI: 76%-95%) 12 months: 87% (CI: 74%-94%) 18 months: 84% (CI: 69%-92%) 24 months: 75% (CI: 51%-89%)	6 months: 96% (CI: 76%-100%) 12 months: 96% (CI: 76%-100%) 18 months: 96% (CI: 76%-100%) 24 months: 96% (CI: 76%-100%)	NR	6 months: 98% (CI: 77%-100%) 12 months: 96% (CI: 75%-100%) 18 months: 96% (CI: 75%-100%) 24 months: 80% (CI: 0%-100%)	NR	NR	NR
Abou Chakra, 2025 (15)	6 months: 84% (CI: 73%-91%) 12 months: 79% (CI: 66%−87%) 24 months: 73% (CI: 59%−83%) 36 months: 68% (CI: 51%−80%)	6 months: 86% (CI: 74%−92%) 12 months: 80% (CI: 67%−88%) 24 months: 75% (CI: 61%−85%) 36 months: 75% (CI: 61%−85%)	6 months: 98% (CI: 89%−100%) 12 months: 97% (CI: 87%−99%) 24 months: 97% (CI: 87%−99%) 36 months: 97% (CI: 87%−99%)	6 months: 100% (CI: 100%−100%) 12 months: 100% (CI: 100%−100%) 24 months: 100% (CI: 100%−100%) 36 months: 100% (CI: 100%−100%)	6 months: 98% (CI: 90%−100%) 12 months: 98% (CI: 90%−100%) 24 months: 85% (CI: 72%−93%) 36 months: 77% (CI: 59%−88%)	6 months: 98% (CI: 89%−100%) 12 months: 97% (CI: 87%−99%) 24 months: 97% (CI: 87%−99%) 36 months: 97% (CI: 87%−99%)	2 (3.07%)	Tumor recurrence, and severe bladder symptoms.
Steinberg, 2020	6 months: 77% (CI: 71%–81%) 12 months: 60% (CI: 54%–66%) 24 months: 46% (CI: 39%–53%)	6 months: 79% (CI: 73%–83%) 12 months: 65% (CI: 59%–71%) 24 months: 52% (CI: 45%–59%)	6 months: 99% (CI: 97%–100%) 12 months: 97% (CI: 94%–99%) 24 months: 93% (CI: 86%–97%)	6 months: 100% (CI: 97%–100%) 12 months: 99% (CI: 97%–100%) 24 months: 96% (CI: 91%–98%)	6 months: 100% (CI: 97%–100%) 12 months: 97% (CI: 94%–99%) 24 months: 87% (CI: 82%–91%)	NR	43 (15.6%)	38 underwent cystectomy for recurrence, 2 were unable to tolerate Induction, 2 for refractory LUTS and 1 was unknown.
Yim, 2023 (17)	6 Months: 74% (CI: 64%–82%) 12 Months: 63% (CI: 52%–72%) 24 Months: 38% (CI: 24%–51%)	6 Months: 78% (CI: 68%–85%) 12 Months: 65% (CI: 54%–75%) 24 Months: 49% (CI: 35%–61%)	6 Months: 93% (CI: 86%–97%) 12 Months: 86% (CI: 77%–92%) 24 Months: 71% (CI: 55%–82%)	6 Months: 100% (CI: 100%–100%) 12 Months: 98% (CI: 91%–99%) 24 Months: 93% (CI: 82%–98%)	6 Months: 100% (CI:100%–100%) 12 Months: 98% (CI: 91%–99%) 24 Months: 92% (CI: 81%–97%)	6 Months: 94% (CI: 87%–97%) 12 Months: 91% (CI: 83%–96%) 24 Months: 79% (CI: 65%–88%)	20 (19.6%)	All patient underwent cystectomy for recurrent disease.
Chevuru, 2023 (18)	12 months: 57% (CI: 46%–66%) 24 months: 44% (CI: 34%–54%) 36 months: 34% (CI: 24%–44%) 60 months: 24% (CI: 15%–34%)	12 months: 60% (CI: 49%–69%) 24 months:50% (CI: 39%–59%) 36 months: 41% (CI: 30%–51%) 60 months: 30% (CI: 20%–41%)	12 months: 96% (CI: 89%–98%) 24 months: 91% (CI: 83%–95%) 36 months: 88% (CI: 79%–93%) 60 months: 82% (CI: 71%–90%)	12 months: 99% (CI: 93%–100%) 24 months: 97% (CI: 90%–99%) 36 months: 93% (CI: 84%–97%) 60 months: 91% (CI: 82%–96%)	12 months: 96% (CI: 89%–98%) 24 months: 87% (CI: 79%–93%) 36 months: 78% (CI: 68%–85%) 60 months: 64% (CI: 52%–74%)	12 months: 89% (CI: 81%–94%) 24 months: 86% (CI: 77%–91%) 36 months: 83% (CI: 73%–89%) 60 months: 75% (CI: 63%–84%)	21 (21.6%)	10 with recurrent NMIBC, 7 recurrent NMIBC after additional salvage therapy, 2 intolerants to Gem/Doce, 1 contracted bladder after perforation, 1 underwent cystectomy at an outside hospital with unknown pathology.
Scilipoti, 2025 (10)	NR	NR	12 months: 96% (CI: 90%–100%)	NR	NR	12 months: 95% (CI: 90%–100%)	5 (6.7%)	High Grade Recurrence

RFS, Recurrence Free Survival; HG RFS, High Grade Recurrence Free Survival; PFS, Progressive Free Survival; CSS, Cancer Specific Survival; OS, Overall Survival; CFS, Cancer Free Survival; NR, Not Reported; LUTS, Lower Urinary Tract Symptoms; NMIBC, Non-Muscle Invasive Bladder Cancer; Gem/Doce, Gemcitabine/Docetaxel; CI, 95% Confidence Interval.

#### 3.4.2 High-grade recurrence-free survival

**Figure 3:**
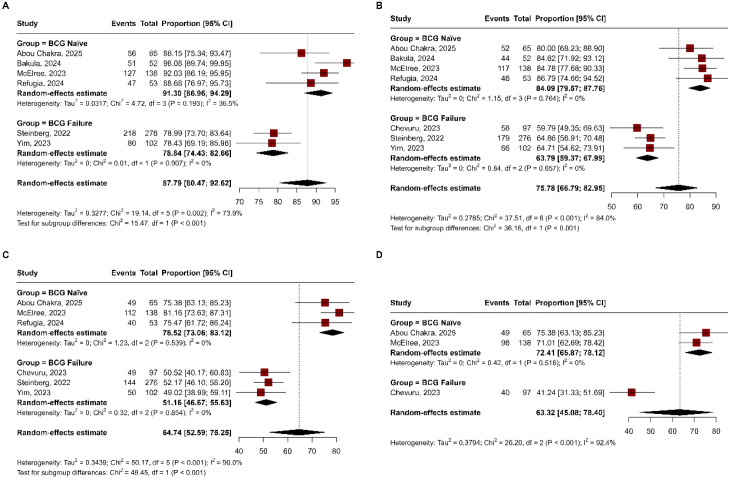
Forest plots of high-grade recurrence-free survival (HG-RFS) at 6 months **(A)**, 12 months **(B)**, 24 months **(C)**, and 36 months **(D)**.

At 6 months, the pooled high-grade recurrence-free survival (HG-RFS) was 87.79% (95% CI 80.47–92.62%), with moderate heterogeneity (I² = 73.9%, τ² = 0.3277). At 12 months, pooled HG-RFS was 75.78% (95% CI 66.79–82.95%), with high heterogeneity (I² = 84.0%, τ² = 0.2785). At 24 months, pooled HG-RFS declined to 64.74% (95% CI 52.59–75.25%), with very high heterogeneity (I² = 90.0%, τ² = 0.3439). At 36 months, pooled HG-RFS was 63.32% (95% CI 45.08–78.40%), with very high heterogeneity (I² = 92.4%, τ² = 0.3794). BCG-naïve patients demonstrated superior HG-RFS at 6 months (91.30%, 95% CI 86.96–94.29%) compared to BCG-failure patients (78.84%, 95% CI 74.43–82.66%), with significant subgroup difference (p < 0.001). At 12 months, BCG-naïve HG-RFS was 84.09% (95% CI 79.57–87.76%) versus 63.79% (95% CI 59.37–67.99%) in BCG failure patients (p < 0.001). At 24 months, BCG-naïve HG-RFS was 78.52% (95% CI 73.06–83.12%) compared to 51.16% (95% CI 46.67–55.63%) in BCG-failure patients (p < 0.001). At 36 months, BCG-naïve HG-RFS was 72.41% (95% CI 65.87–78.12%) versus 41.24% (95% CI 31.33–51.69%) in BCG-failure patients (**Figure 3**).

#### 3.4.3 Progression-free survival

**Figure 4:**
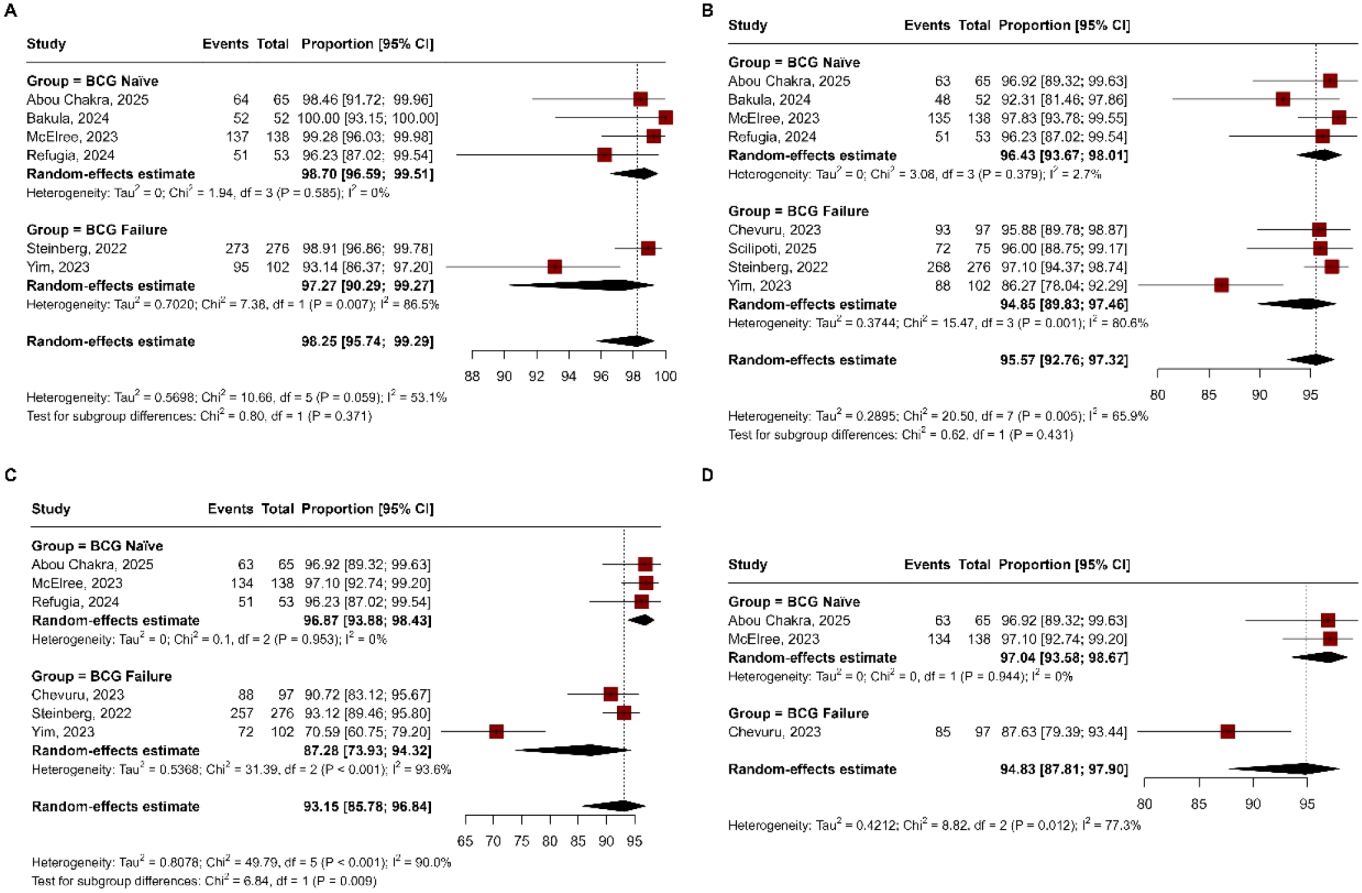
Forest plots of progression-free survival (PFS) at 6 months **(A)**, 12 months **(B)**, 24 months **(C)**, and 36 months **(D)**.

At 6 months, the pooled progression-free survival (PFS) was 98.25% (95% CI 95.74 99.29%), with moderate heterogeneity (I² = 53.1%, τ² = 0.5698). At 12 months, pooled PFS was 95.57% (95% CI 92.76–97.32%), with moderate heterogeneity (I² = 65.9%, τ² = 0.2895). At 24 months, pooled PFS was 93.15% (95% CI 85.78–96.84%), with very high heterogeneity (I² = 90.0%, τ² = 0.8078). At 36 months, pooled PFS was 94.83% (95% CI 87.81–97.90%), with high heterogeneity (I² = 77.3%, τ² = 0.4212). BCG-naïve and BCG-failure patients demonstrated comparable PFS across all time points with a significant subgroup difference emerging at 24 months. At 6 months, BCG-naïve PFS was 98.70% (95% CI 96.59–99.51%) versus 97.27% (95% CI 90.29–99.27%) in BCG failure patients (p = 0.371). At 12 months, BCG-naïve PFS was 96.43% (95% CI 93.67 98.01%) compared to 94.85% (95% CI 89.83–97.46%) in BCG-failure patients (p = 0.431). At 24 months, BCG-naïve PFS was 96.87% (95% CI 93.88–98.43%) versus 87.28% (95% CI 73.93–94.32%) in BCG-failure patients (p = 0.009). At 36 months, BCG-naïve PFS was 97.04% (95% CI 93.58–98.67%) versus 87.63% (95% CI 79.39 93.44%) in BCG-failure patients (**Figure 4**).

#### 3.4.4 Cancer-specific Survival

**Figure 5:**
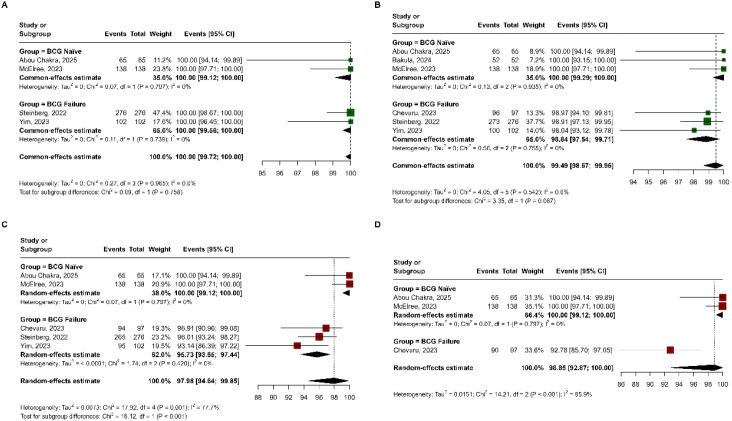
Forest plots of cancer-specific survival (CSS) at 6 months **(A)**, 12 months **(B)**, 24 months **(C)**, and 36 months **(D)**.

At 6 months, the pooled cancer-specific survival (CSS) was 100.0% (95% CI 99.72 100.00%), with no heterogeneity (I² = 0.0%, τ² = 0). At 12 months, pooled CSS was 99.49% (95% CI 98.67–99.96%), with no heterogeneity (I² = 0.0%, τ² = 0). At 24 months, pooled CSS was 97.98% (95% CI 94.64–99.85%), with significant heterogeneity (I² = 77.7%, τ² ≈ 0.0073). At 36 months, pooled CSS was 98.85% (95% CI 92.87–100.00%), with significant heterogeneity (I² = 85.9%, τ² ≈ 0.0151). BCG-naïve patients demonstrated comparable or slightly superior CSS at all time points without significant subgroup differences at 6 and 12 months. At 6 months, both BCG-naïve and BCG-failure cohorts achieved 100.0% CSS (p = 0.758). At 12 months, BCG-naïve CSS was 100.00% (95% CI 99.29–100.00%) versus 98.84% (95% CI 97.54–99.71%) in BCG-failure patients (p = 0.067). At 24 months, BCG-naïve CSS was 100.00% (95% CI 99.12–100.00%) compared to 95.73% (95% CI 93.65–97.44%) in BCG-failure patients (p < 0.001). At 36 months, BCG-naïve CSS was 100.00% (95% CI 99.12–100.00%) versus 92.78% (95% CI 85.70–97.05%) in BCG-failure patients (**Figure 5**).

#### 3.4.5 Overall survival

**Figure 6:**
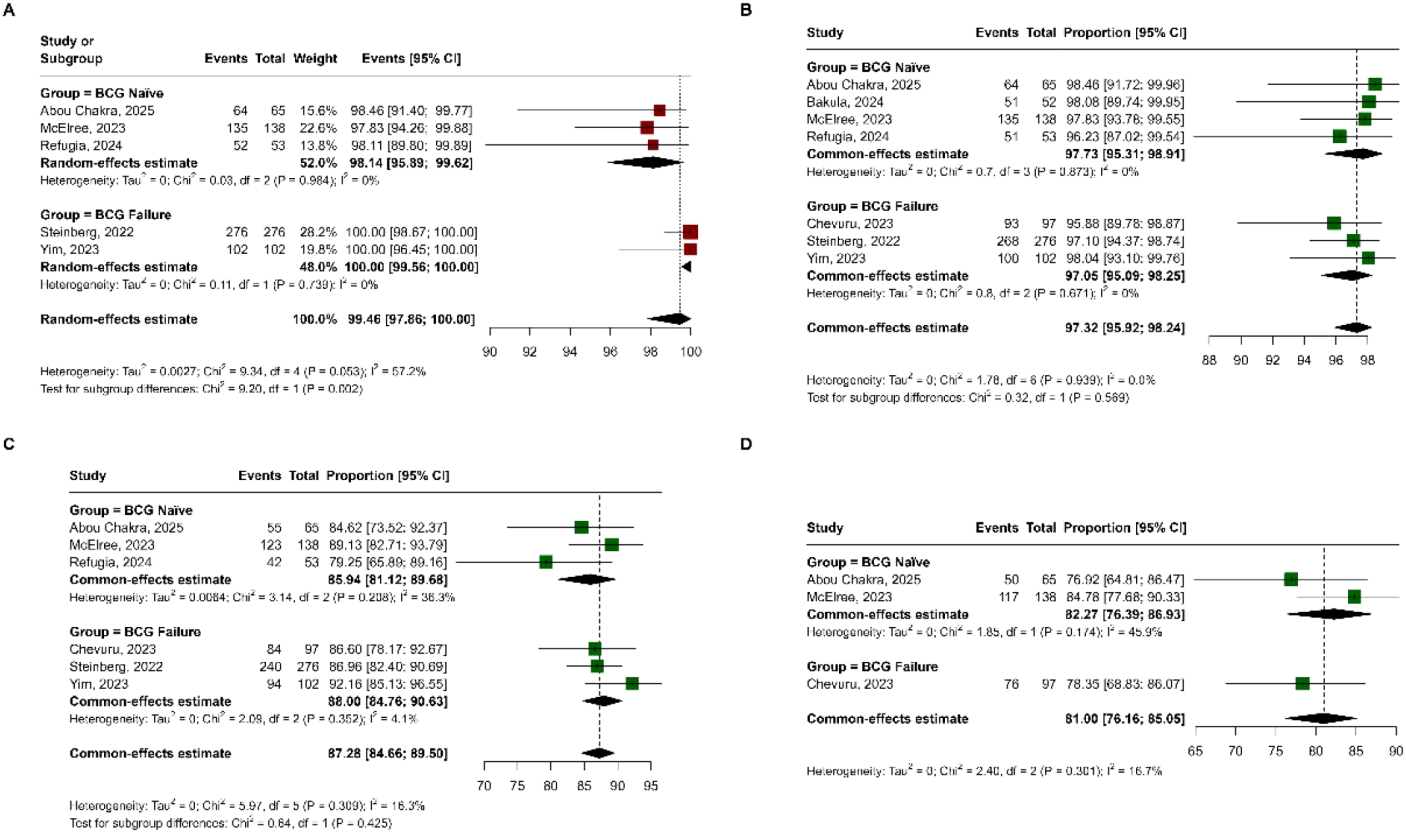
Forest plots of overall survival (OS) at 6 months **(A)**, 12 months **(B)**, 24 months **(C)**, and 36 months **(D)**.

At 6 months, the pooled overall survival (OS) was 99.46% (95% CI 97.86–100.0%), with significant heterogeneity (I² = 57.2%, τ² = 0.0027). At 12 months, pooled OS was 97.32% (95% CI 95.92–98.24%), with no heterogeneity (I² = 0.0%, τ² = 0). At 24 months, pooled OS was 87.28% (95% CI 84.66–89.50%), with low heterogeneity (I² = 16.3%, τ² ≈ 0). At 36 months, pooled OS was 81.00% (95% CI 76.16–85.05%), with low heterogeneity (I² = 16.7%, τ² ≈ 0). BCG-naïve and BCG-failure patients demonstrated comparable OS across all time points without significant subgroup differences. At 6 months, BCG-naïve OS was 98.14% (95% CI 95.89–99.62%) versus 100.00% (95% CI 99.56–100.00%) in BCG failure patients (p = 0.002). At 12 months, BCG-naïve OS was 97.73% (95% CI 95.31 98.91%) compared to 97.05% (95% CI 95.09–98.25%) in BCG-failure patients (p = 0.569). At 24 months, BCG-naïve OS was 85.94% (95% CI 81.12–89.68%) versus 88.00% (95% CI 84.76–90.63%) in BCG-failure patients (p = 0.425). At 36 months, BCG-naïve OS was 82.27% (95% CI 76.39–86.93%) versus 78.35% (95% CI 68.83% 86.07%) in BCG-failure patients (**Figure 6**).

#### 3.4.6 Cystectomy-free survival

**Figure 7:**
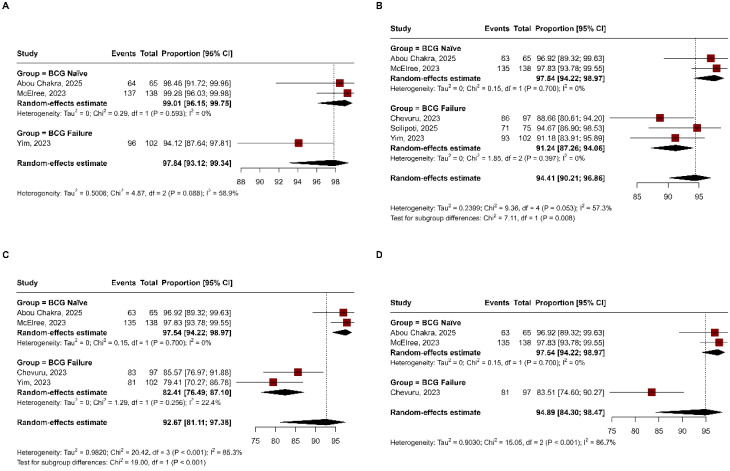
Forest plots of cystectomy-free survival (CFS) at 6 months **(A)**, 12 months **(B)**, 24 months **(C)**, and 36 months **(D)**.

At 6 months, the pooled cystectomy-free survival (CFS) was 97.84% (95% CI 93.12 99.34%), with moderate heterogeneity (I² = 58.9%, τ² = 0.5008). At 12 months, pooled CFS was 94.41% (95% CI 90.21–96.86%), with moderate heterogeneity (I² = 57.3%, τ² = 0.2399). At 24 months, pooled CFS was 92.67% (95% CI 81.11–97.38%), with high heterogeneity (I² = 85.3%, τ² = 0.9820). At 36 months, pooled CFS was 94.89% (95% CI 84.30–98.47%), with high heterogeneity (I² = 86.7%, τ² = 0.9030). BCG-naïve patients demonstrated superior CFS at 6 months (99.01%, 95% CI 96.15 99.75%) compared to BCG-failure patients (94.12%, 95% CI 87.64–97.81%). At 12 months, BCG-naïve CFS was 97.54% (95% CI 94.22–98.97%) versus 91.24% (95% CI 87.26–94.06%) in BCG-failure patients (p = 0.008). At 24 months, BCG-naïve CFS was 97.54% (95% CI 94.22–98.97%) compared to 82.41% (95% CI 76.49–87.10%) in BCG-failure patients (p < 0.001). At 36 months, BCG-naïve CFS was 97.54% (95% CI 94.22–98.97%) versus 83.51% (95% CI 74.60–90.27%) in BCG-failure patients (**Figure 7**).

### 3.5 Safety and adverse events

#### 3.5.1 Incidence of adverse events

**Figure 8:**
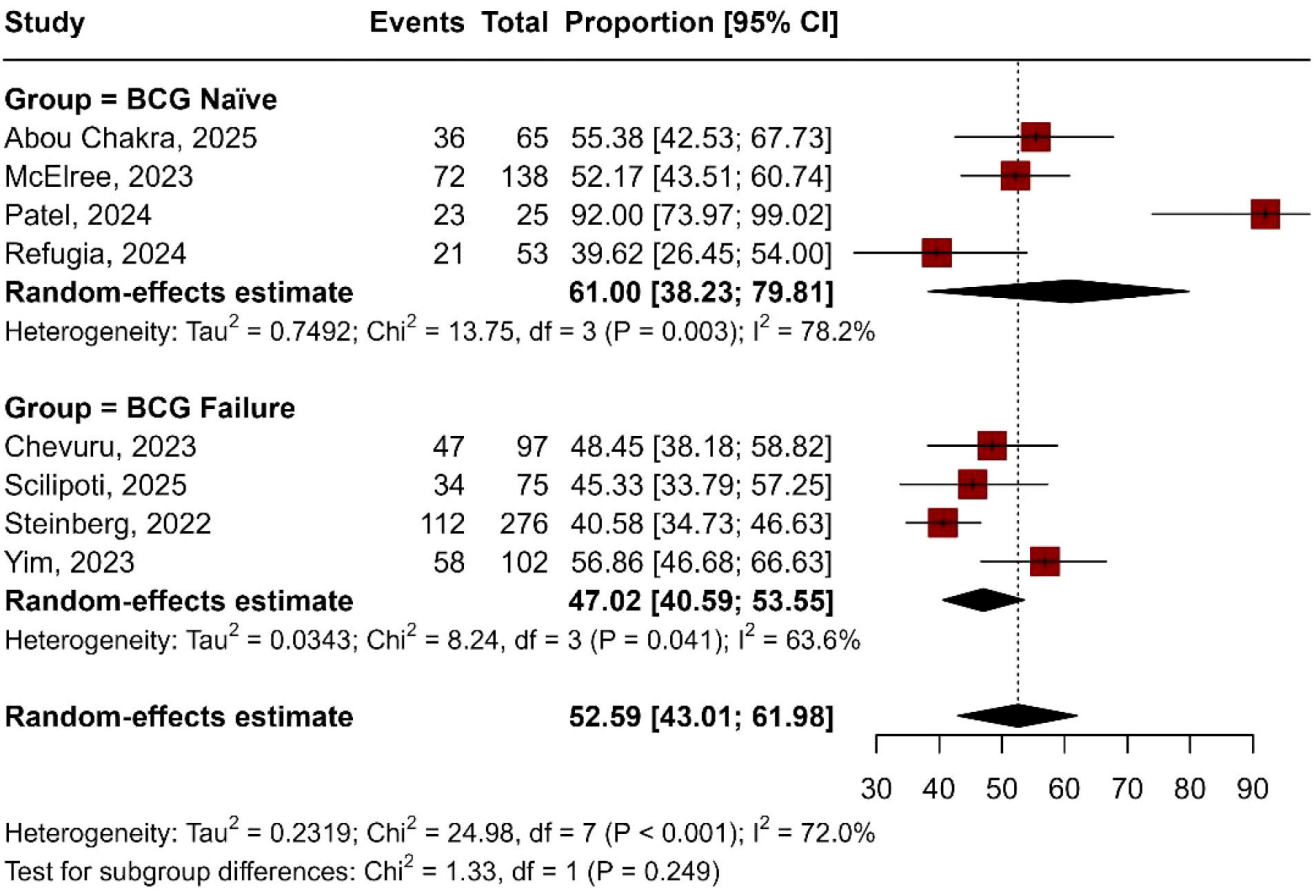
Forest plot of the pooled proportion of patients experiencing treatment-related adverse events.

The pooled proportion of patients experiencing at least one treatment-related adverse event was 52.59% (95% CI 43.01–61.98%), with high heterogeneity (I² = 72.0%, τ² = 0.2319). BCG-naïve patients demonstrated a higher incidence of adverse events with 61.00% (95% CI 38.23–79.81%) compared to BCG-failure patients which was 47.02% (95% CI 40.59–53.55%); however, this difference was not statistically significant (p = 0.249) (**Figure 8**, **Table 4**).

**Table 4:** Treatment-Related Adverse Events and Safety.

Author, year	Number of patient reporting adverse event	Details of adverse event	Unable to tolerate	Affected treatment schedule
McElree, 2023	72 (52.2%)	Bladder Spasm = 29 (32.5%) UTI = 13 (14.6%) Urinary frequency/urgency = 12 (13.4%) Hematuria = 8 (8.9%) Dysuria = 7 (7.8%) Fatigue/Flulike symptoms = 7 (7.8%) Nausea = 5 (5.6%) Urinary retention = 3 (3.3%) Bladder pain = 3 (3.3%) Rash = 1 (1.1%) Syncope = 1 (1.1%)	4 (2.9 %)	NR
Patel, 2024 (12)	23 (92%)	UTI = 10 (16.9%) Fatigue = 8 (13.5%) Dysuria/Suprapubic Pain = 7 (11.8%) Respiratory Infection = 6 (10.1%) Hematuria = 5 (8.4%) Nausea/Vomiting/Gastrointestinal Symptoms = 5 (8.4%) Urinary frequency/urgency = 4 (6.7%) Vision Changes/ Retinopathy = 3 (5%) Thrombocytopenia/Leukopenia = 2 (3.3%) Vasovagal Syncope = 2 (3.3%) Tremor = 2 (3.3%) Headache = 2 (3.3%) Gait Disturbance = 1 (1.6%) Altered Mental Status = 1 (1.6%) Skin or Mucosal Rashes or Lesions = 1 (1.6%)	1 (4%)	3 (12%)
Bakula, 2024 (11)	NR	NR	NR	NR
Refugia, 2024 (14)	21 (40%)	Cystitis, non-infective: 14 (60.9%) UTI: 4 (17.4%) Bladder spasm: 3 (13%) Hematuria: 2 (8.7%)	NR	10 (18.8%)
Abou Chakra, 2025 (15)	36 (55.4%)	Bladder spasms: 15 (31.9%) UTI: 9 (19.1%) Frequency: 7 (14.9%) Hematuria: 4 (8.5%) Bladder pain: 3 (6.4%) Fatigue/flu-like symptoms: 3 (6.4%) Dysuria: 2 (4.3%) Nausea: 2 (4.3%) Urinary Retention: 2 (4.3%)	2 (3.1%)	NR
Steinberg, 2020	112 (40.6%)	Urinary urgency/frequency = 61 (44.5%) Dysuria = 43 (31.4%) Hematuria =29 (21.2%) Urinary retention = 4 (2.9%)	9 (3.3%)	26 (9.4%)
Yim, 2023 (17)	58 (56.9%)	Urinary urgency/frequency = 42 (53.8%) Dysuria = 21 (26.9%) Hematuria = 8 (10.3%) UTI = 6 (7.7%) Urinary retention = 1 (1.3%)	NR	7 (6.9%)
Chevuru, 2023 (18)	47 (48%)	Bladder Spasm: 15 (19.2%) Urinary Frequency/Urgency: 12 (15.4%) UTI: 11 (14.1%) Dysuria: 9 (11.5%) Hematuria: 8 (10.3%) Fatigue: 7 (9%) Nausea: 6 (7.7%) Bladder/Flank Pain: 4 (5.1%) Skin Irritation: 3 (3.9%) Flu-Like Symptoms: 2 (2.6%) Urinary Retention: 1 (1.3%)	2 (2.1%)	13 (13%)
Scilipoti, 2025 (10)	34 (45%)	Irritative LUTS: 23 (46.9%) Asthenia: 7 (14.3%) Hematuria: 6 (12.2%) Fever: 6 (12.2%) UTI: 3 (6.1%) Nausea: 3 (6.1%) Headache: 1 (2%)	6 (8.0%)	NR

NR, Not Reported; UTI, Urinary Tract Infection; LUTS, Lower Urinary Tract Symptoms.

#### 3.5.2 Treatment tolerability

**Figure 9:**
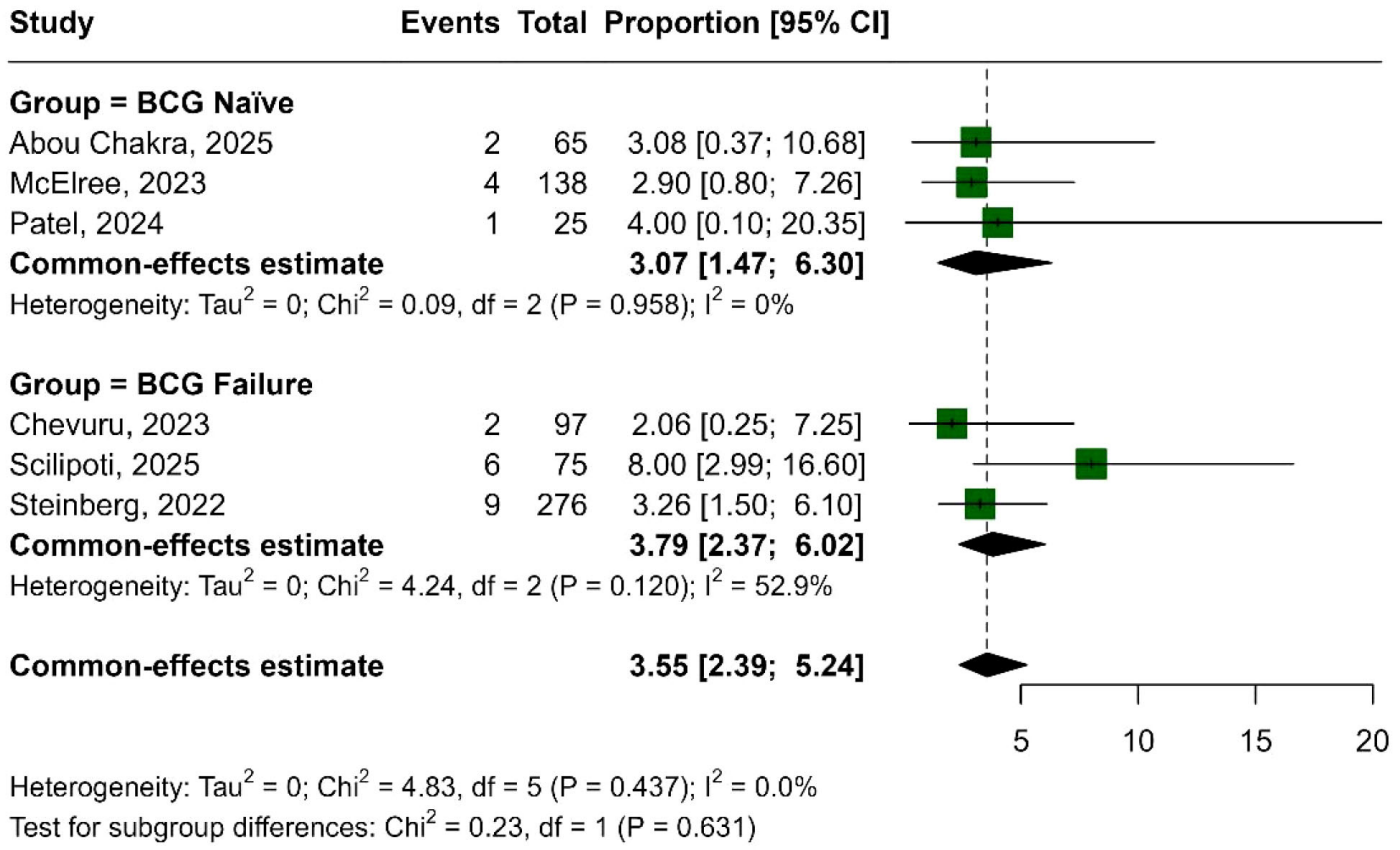
Forest plot of the pooled proportion of patients unable to tolerate treatment.

The pooled proportion of patients unable to tolerate treatment was 3.55% (95% CI 2.39–5.24%), with no heterogeneity (I² = 0.0%, τ² = 0). BCG-naïve patients demonstrated a comparable incidence with 3.07% (95% CI 1.47–6.30%) to BCG-failure patients which was 3.79% (95% CI 2.37–6.02%), with no significant subgroup difference (p = 0.631) (**Figure 9**).

#### 3.5.3 Adverse events affecting treatment schedule

**Figure 10:**
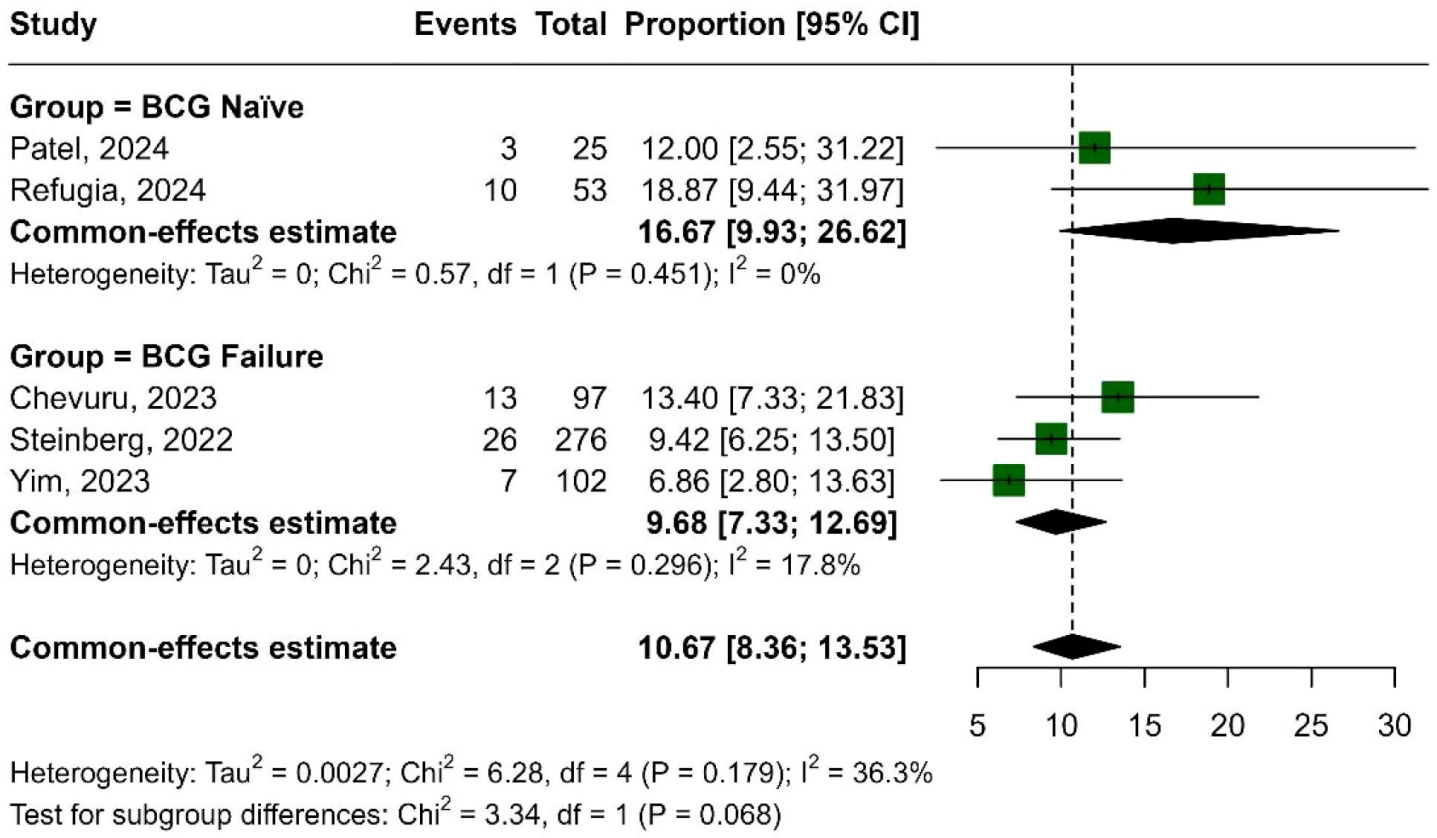
Forest plot of the pooled proportion of patients with adverse events affecting treatment schedule.

The pooled proportion of patients with adverse events affecting treatment schedule was 10.67% (95% CI 8.36–13.53%), with low heterogeneity (I² = 36.3%, τ² = 0.0027). BCG-naïve patients demonstrated a higher incidence with 16.67% (95% CI 9.93–26.62%) compared to BCG-failure patients which was 9.68% (95% CI 7.33–12.69%), though this difference approached but did not reach statistical significance (p = 0.068) (**Figure 10**).

#### 3.5.4 Hematuria

**Figure 11:**
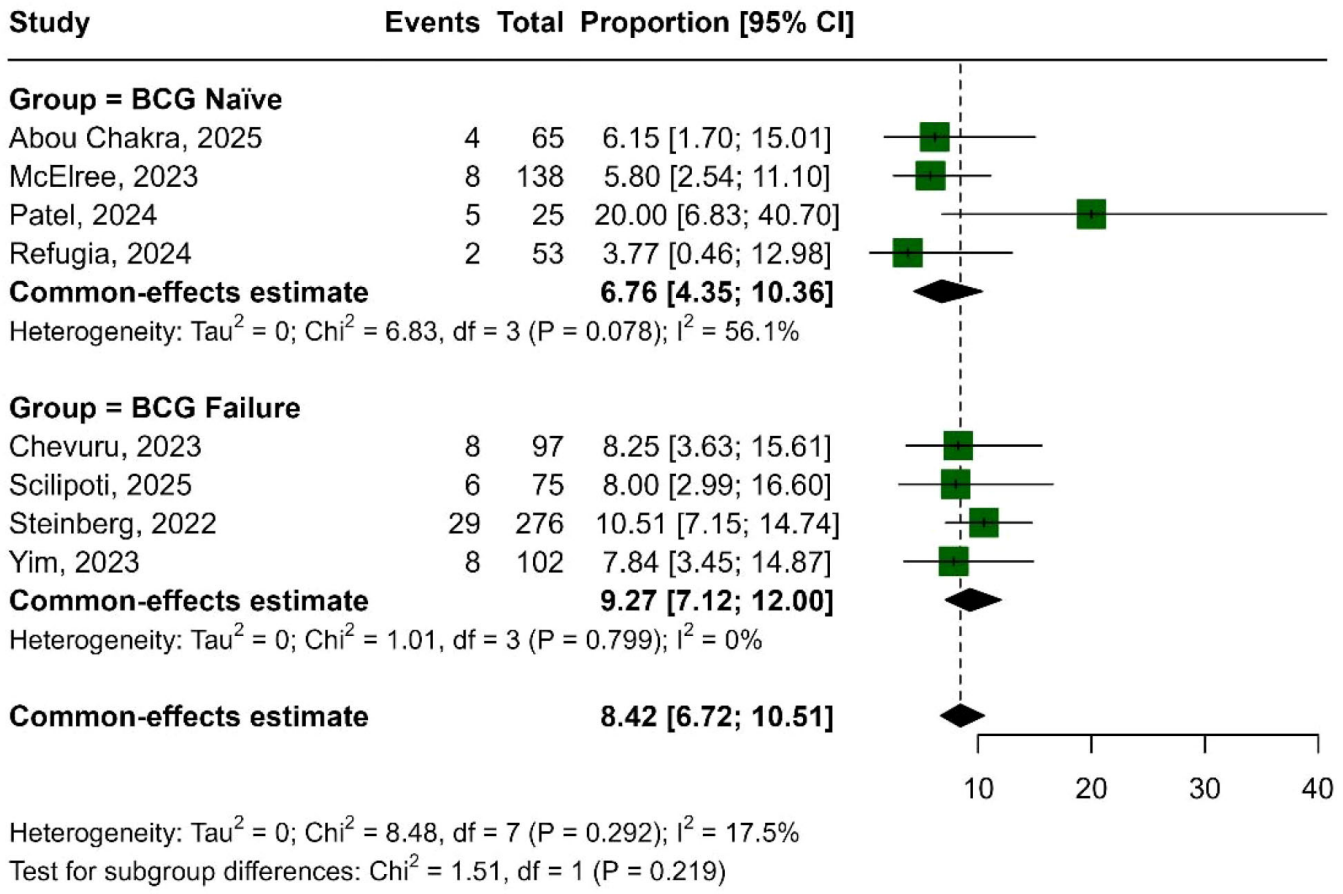
Forest plot of the pooled proportion of patients experiencing hematuria.

The pooled proportion of patients experiencing hematuria was 8.42% (95% CI 6.72–10.51%), with low heterogeneity (I² = 17.5%, τ² = 0). BCG-naïve patients demonstrated a comparable incidence with 6.76% (95% CI 4.35–10.36%) to BCG-failure patients which was 9.27% (95% CI 7.12–12.00%), with no significant subgroup difference (p = 0.219) (**Figure 11**).

#### 3.5.5 Urinary tract infection

**Figure 12:**
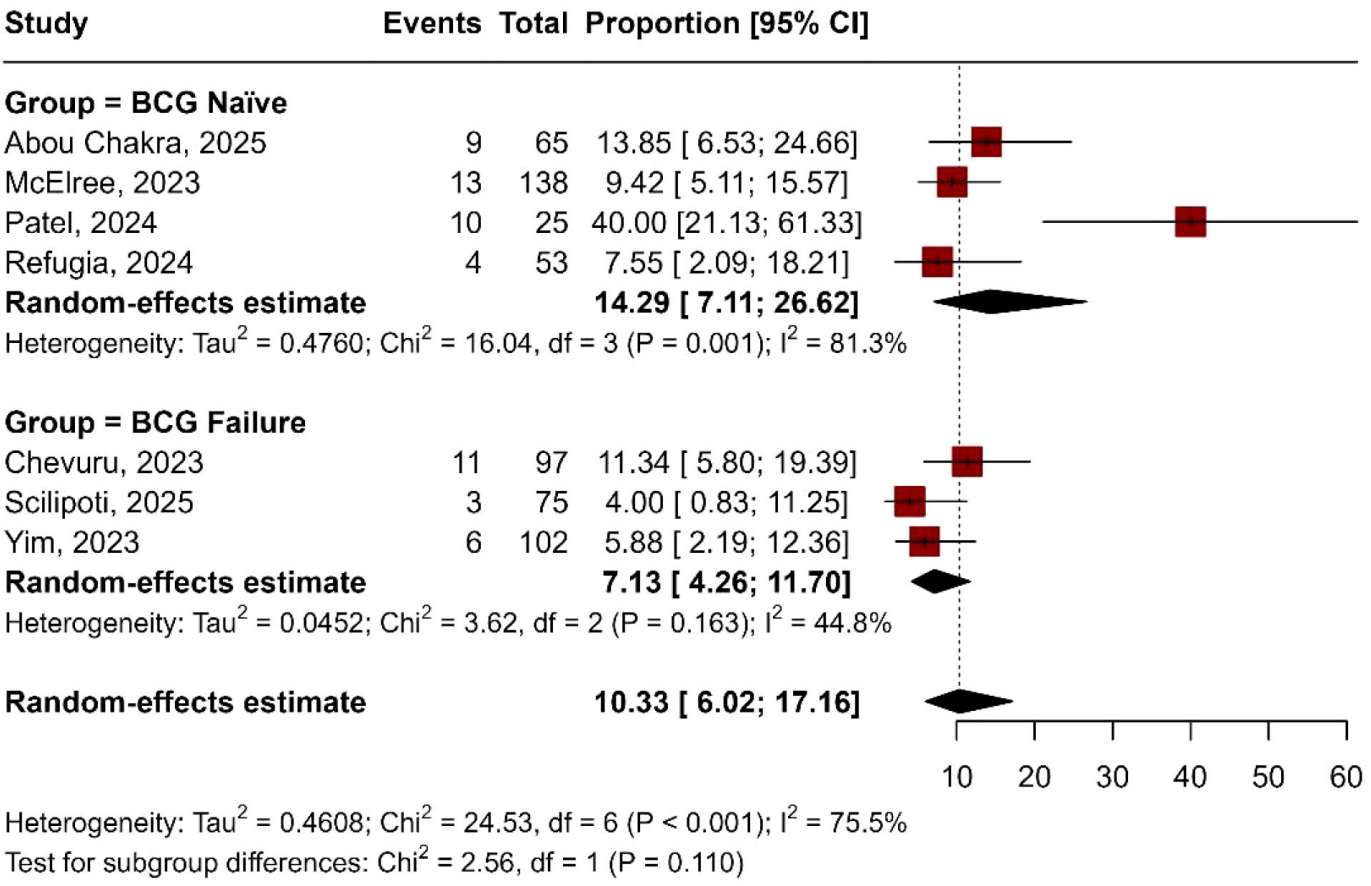
Forest plot of the pooled proportion of patients experiencing urinary tract infection.

The pooled proportion of patients experiencing urinary tract infection was 10.33% (95% CI 6.02–17.16%), with high heterogeneity (I² = 75.5%, τ² = 0.4608). BCG-naïve patients demonstrated a higher incidence with 14.29% (95% CI 7.11–26.62%) compared to BCG-failure patients which was 7.13% (95% CI 4.26–11.70%), though this difference was not statistically significant (p = 0.110) (**Figure 12**).

#### 3.5.6 Urinary frequency/urgency

**Figure 13:**
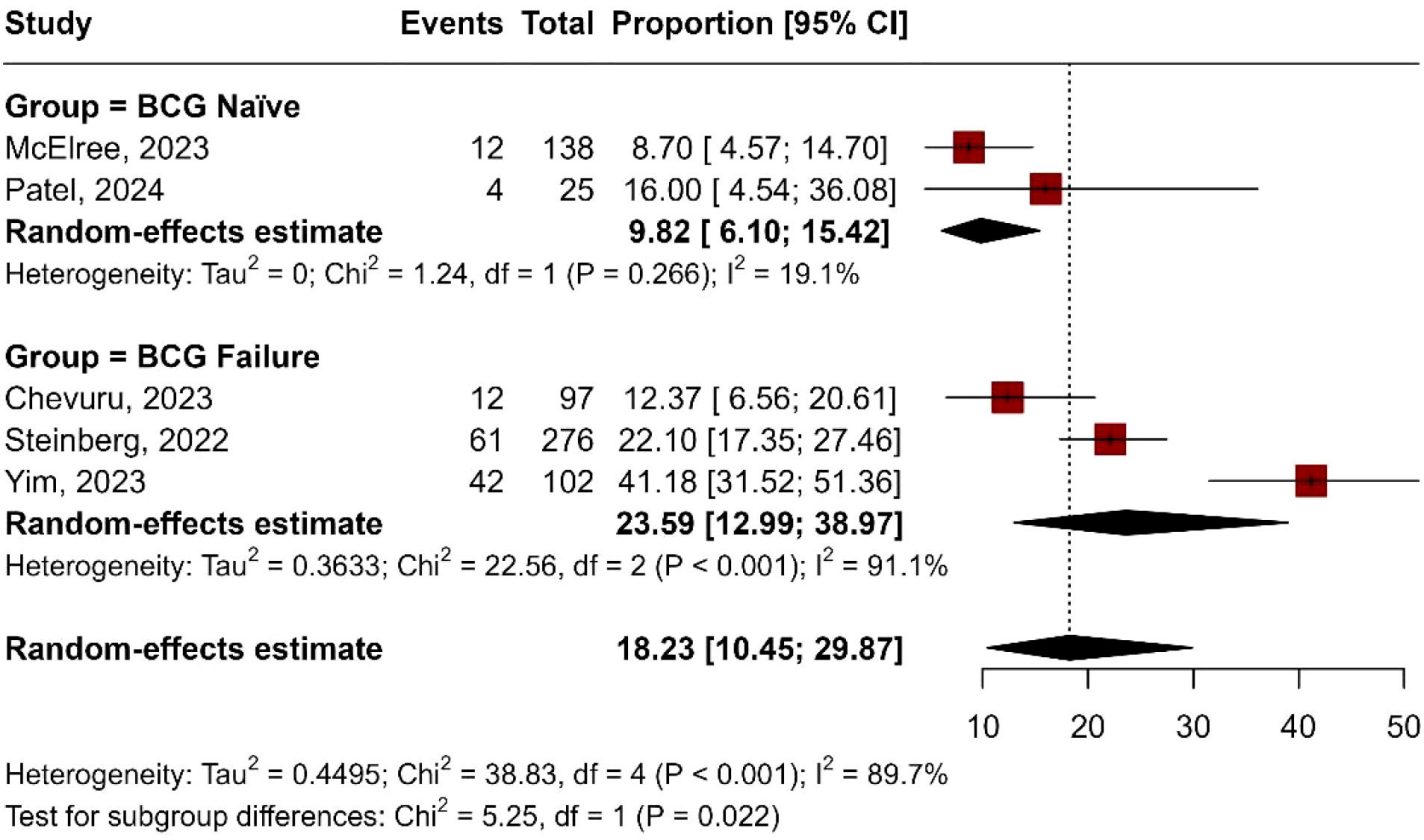
Forest plot of the pooled proportion of patients experiencing urinary frequency or urgency.

The pooled proportion of patients experiencing urinary frequency or urgency was 18.23% (95% CI 10.45–29.87%), with very high heterogeneity (I² = 89.7%, τ² = 0.4495). BCG-naïve patients demonstrated a significantly lower incidence with 9.82% (95% CI 6.10–15.42%) compared to BCG-failure patients which was 23.59% (95% CI 12.99–38.97%), with statistically significant subgroup difference (p = 0.022) (**Figure 13**).

#### 3.5.7 Dysuria

**Figure 14:**
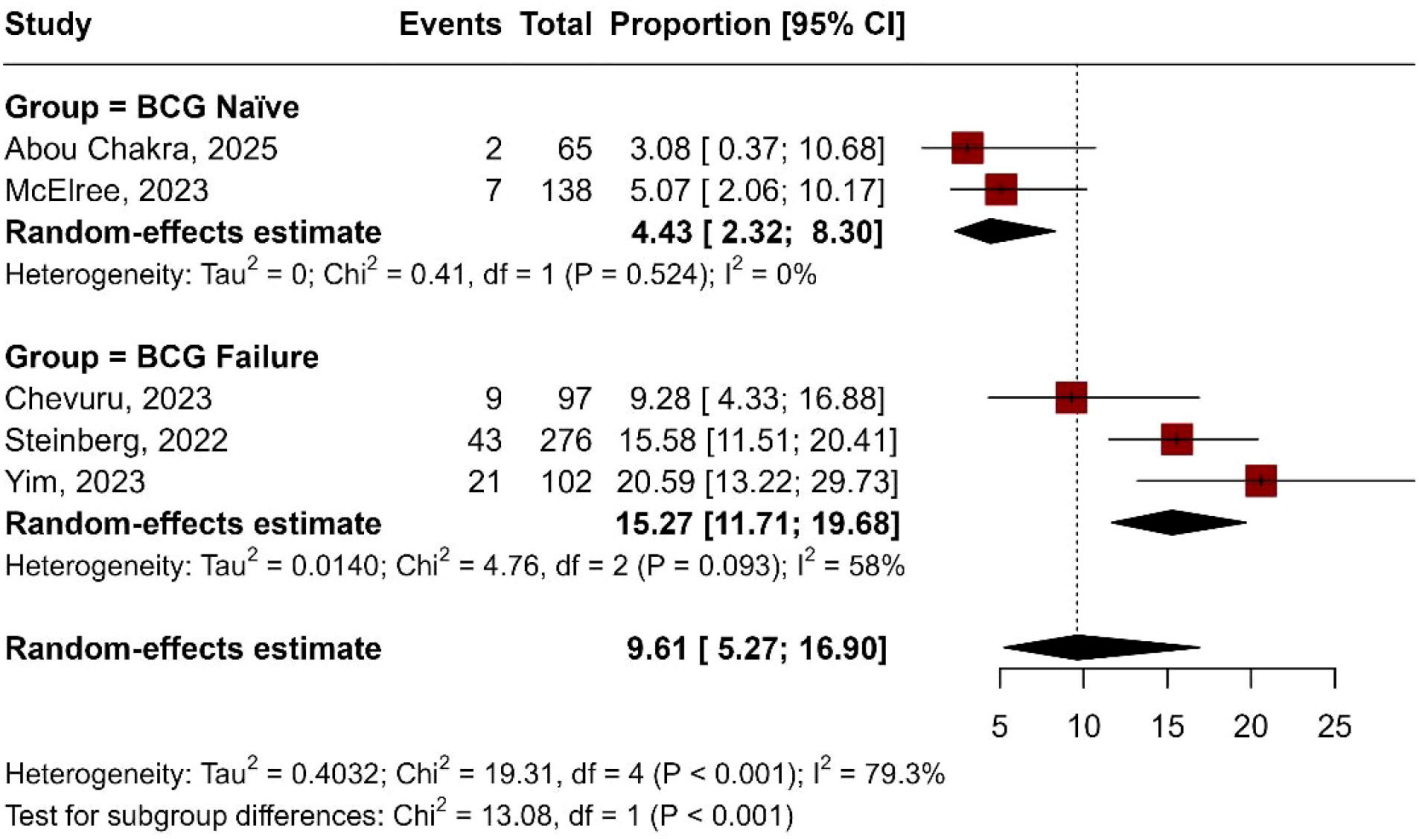
Forest plot of the pooled proportion of patients experiencing dysuria.

The pooled proportion of patients experiencing dysuria was 9.61% (95% CI 5.27–16.90%), with high heterogeneity (I² = 79.3%, τ² = 0.4032). BCG-naïve patients demonstrated a significantly lower incidence with 4.43% (95% CI 2.32–8.30%) compared to BCG-failure patients which was 15.27% (95% CI 11.71–19.68%), with statistically significant subgroup difference (p < 0.001) (**Figure 14**).

#### 3.5.8 Urinary retention

**Figure 15:**
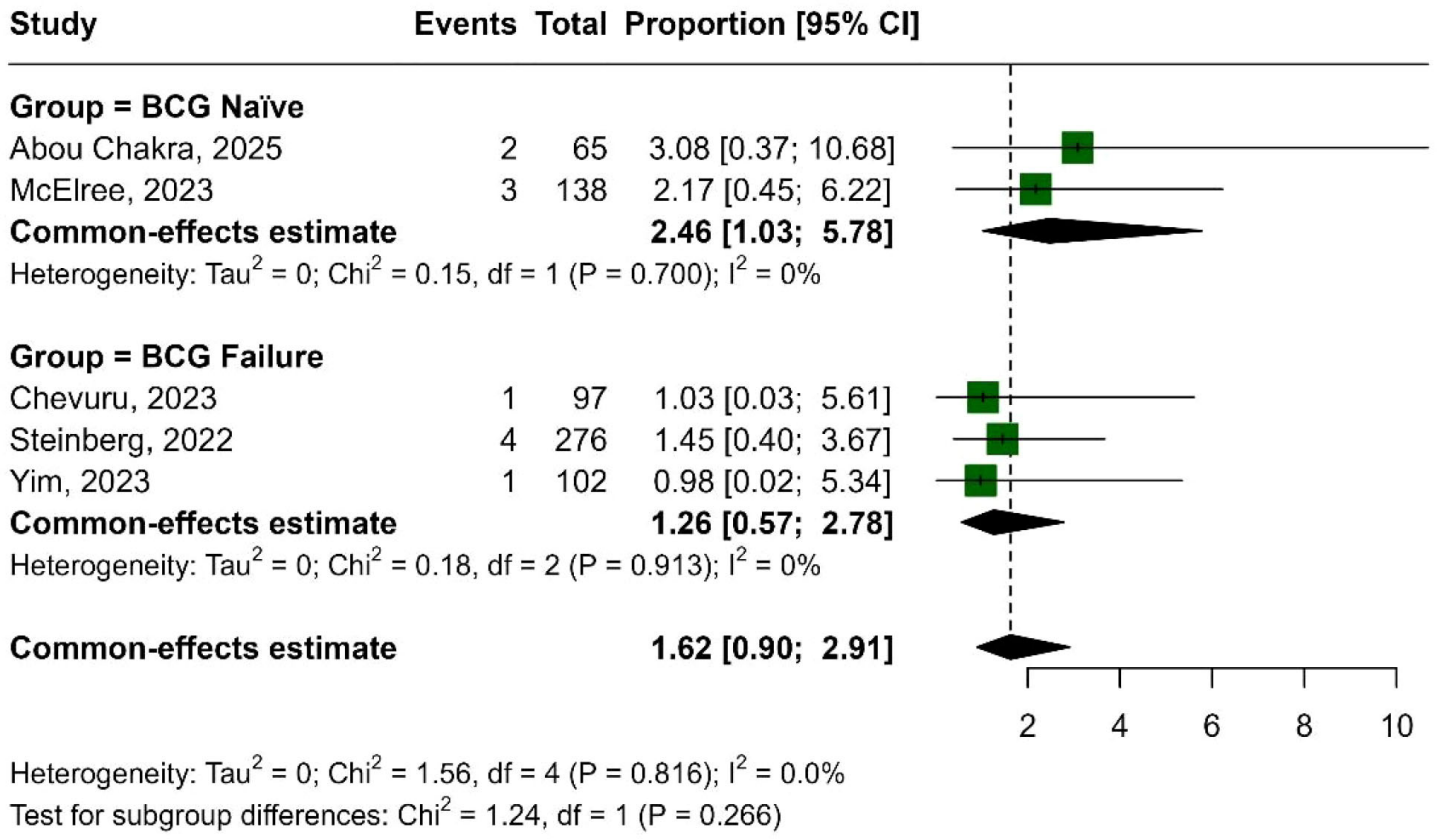
Forest plot of the pooled proportion of patients experiencing urinary retention.

The pooled proportion of patients experiencing urinary retention was 1.62% (95% CI 0.90–2.91%), with no heterogeneity (I² = 0.0%, τ² = 0). BCG-naïve patients demonstrated a slightly higher incidence with 2.46% (95% CI 1.03–5.78%) compared to BCG-failure patients which was 1.26% (95% CI 0.57–2.78%), though this difference was not statistically significant (p = 0.266) (**Figure 15**).

#### 3.5.9 Nausea

**Figure 16:**
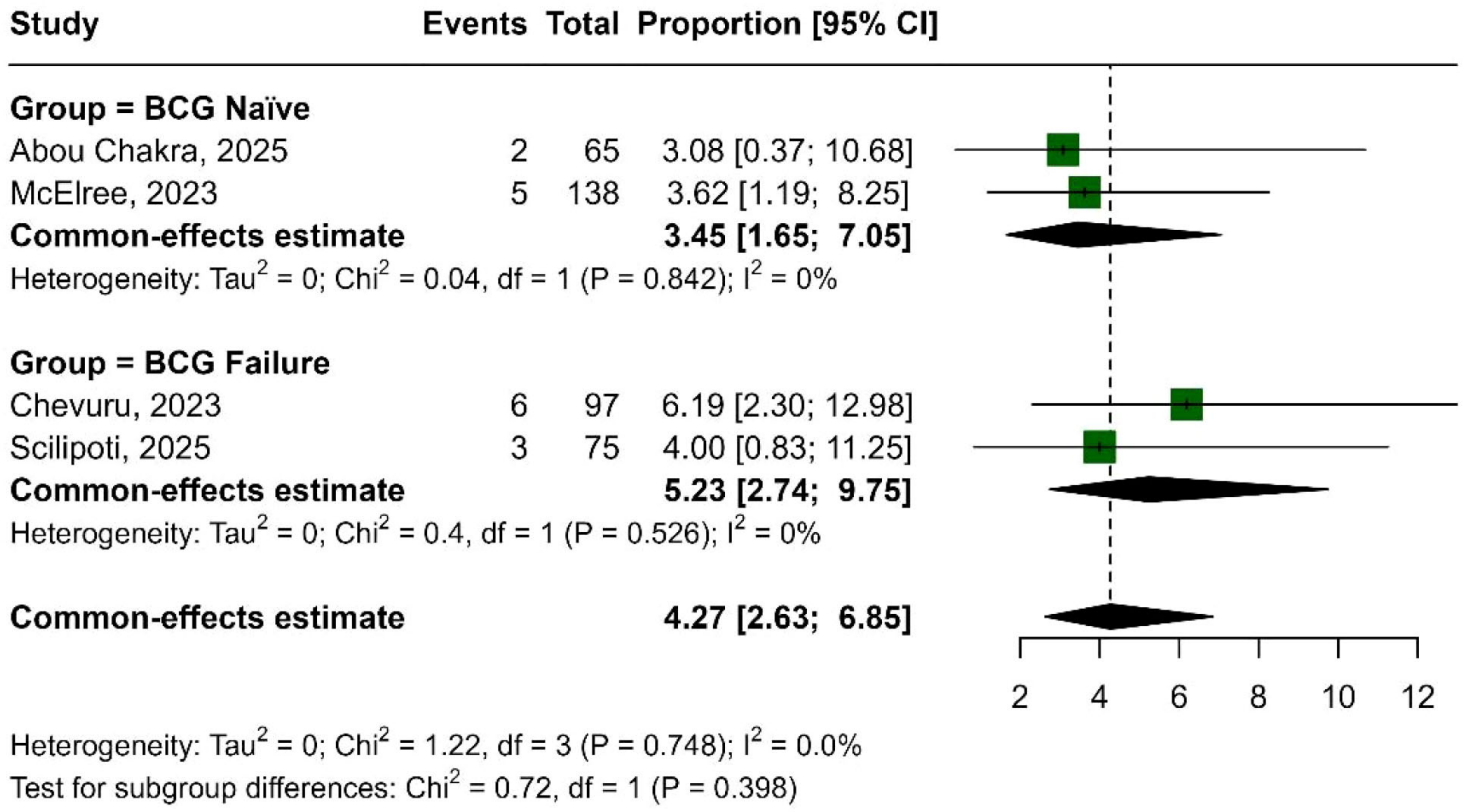
Forest plot of the pooled proportion of patients experiencing nausea.

The pooled proportion of patients experiencing nausea was 4.27% (95% CI 2.63–6.85%), with no heterogeneity (I² = 0.0%, τ² = 0). BCG-naïve patients demonstrated a slightly lower incidence with 3.45% (95% CI 1.65–7.05%) compared to BCG-failure patients which was 5.23% (95% CI 2.74–9.75%), though this difference was not statistically significant (p = 0.398) (**Figure 16**).

#### 3.5.10 Bladder spasm

**Figure 17:**
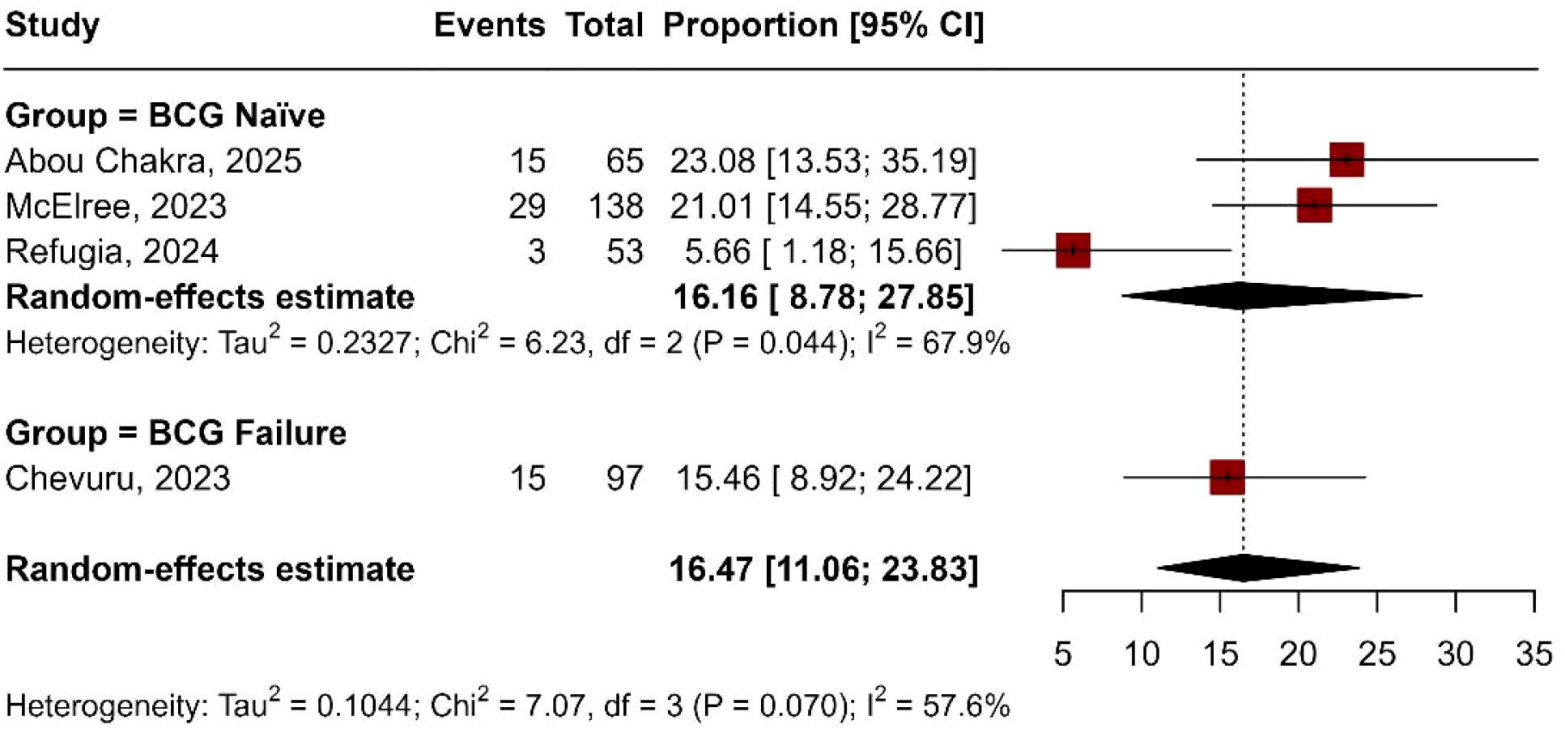
Forest plot of the pooled proportion of patients experiencing bladder spasm.

The pooled proportion of patients experiencing bladder spasm was 16.47% (95% CI 11.06–23.83%), with moderate heterogeneity (I² = 57.6%, τ² = 0.1044). BCG-naïve patients demonstrated a comparable incidence (16.16%, 95% CI 8.78–27.85%) to BCG-failure patients (15.46%, 95% CI 8.92–24.22%) (**Figure 17**).

## 4 Discussion

In the contemporary management of high- and very high-risk NMIBC, treatment increasingly centers on bladder-preserving intravesical strategies for BCG-naïve and BCG-failure patients who wish to avoid, defer, or are unfit for radical cystectomy. Within this context, recent European Association of Urology recommendations outline several non-BCG intravesical or locoregional options, one of which is optimized single-agent chemotherapy. Intravesical mitomycin C or gemcitabine is one example, delivered with prolonged dwell times or device-assisted hyperthermia. Other options include sequential or combination chemotherapy regimens such as gemcitabine plus docetaxel or gemcitabine plus mitomycin C, and novel agents like the viral vector–based nadofaragene firadenovec. For BCG-unresponsive patients, systemic checkpoint inhibition such as pembrolizumab may be used if bladder preservation is desired. Despite this expanding armamentarium, many of these approaches are limited by cost, availability, technical requirements, or immature long-term data, and there remains a clear need for accessible, well-tolerated intravesical regimens that can be applied across both BCG-naïve and BCG-failure high-risk populations. Against this backdrop, sequential intravesical GEM/DOCE has emerged as a pragmatic chemotherapy combination, with our systematic review demonstrating meaningful oncologic control in high- and very high-risk NMIBC across both BCG-naïve and BCG-failure populations (19).

Across nine studies, GEM/DOCE was associated with favorable short- and intermediate-term recurrence-free and high-grade recurrence-free outcomes, with consistently preserved progression-free survival, CSS, and OS. Additionally, CFS remained high across follow-up, supporting the role of GEM/DOCE as an effective bladder-preserving strategy in aggressive NMIBC. In this context, RFS decreased with increasing follow-up, in keeping with the expected course of high-risk NMIBC. HG-RFS also declined over time but remained consistently higher than overall RFS at corresponding time points. Progression to muscle-invasive disease was infrequent, with PFS exceeding 90% at both 12 and 24 months in most cohorts. CSS remained high at both 12 and 24 months, exceeding 95% at 24 months and remaining stable at longer follow-up, supporting sustained oncologic control. OS demonstrated a gradual decline from 12 to 24 months and beyond, likely influenced by patient age and comorbidity rather than bladder cancer–related mortality. Notably, CFS remained high at 12 and 24 months, indicating that bladder preservation was achievable for a substantial proportion of patients despite disease recurrence (10–18).

When stratified by BCG status, BCG-naïve patients consistently demonstrated superior oncologic outcomes, including higher recurrence-free and HG-RFS, compared with BCG-failure cohorts. PFS, CSS, OS, and CFS also remained more favorable in BCG-naïve populations across follow-up (10–18). These differences are biologically plausible and reflect the more aggressive tumor behavior and relative treatment resistance characteristic of previously treated disease. Nevertheless, even among BCG-failure cohorts, GEM/DOCE achieved clinically meaningful disease control, with low rates of progression to muscle-invasive disease, preserved CSS, and acceptable bladder-preservation outcomes, supporting its role as a salvage intravesical option in carefully selected patients (10, 16–18). GEM/DOCE was generally well tolerated across the included studies. Treatment-related adverse events were common but were predominantly mild to moderate and largely confined to the urinary tract. The most frequently reported events included urinary urgency or frequency, bladder spasms, dysuria, hematuria, and urinary tract infections. Systemic adverse events were uncommon and typically mild, with fatigue, flu-like symptoms, and gastrointestinal complaints reported at low rates. Serious systemic toxicity was rare (10, 12–18). Importantly, treatment discontinuation due to intolerance was uncommon, occurring in fewer than 5% of patients in most cohorts, whereas treatment schedule modifications were more frequently required, though still limited to a relatively small proportion of patients. Most patients were able to complete induction therapy, and a substantial proportion proceeded to maintenance treatment. Taken together, these findings indicate a favorable balance between oncologic efficacy and tolerability, supporting the role of GEM/DOCE as a bladder-preserving alternative treatment option for patients with high-risk NMIBC (10, 12–18). In a comparative study of intravesical GEM/DOCE and BCG for high-risk NMIBC, GEM/DOCE was associated with higher recurrence-free and HG-RFS across all evaluated time points. At 6 months, RFS was 90% with GEM/DOCE compared with 72% with BCG, representing an 18% absolute difference, and this discrepancy persisted at 12 months, with rates of 83% versus 67%. At 24 months, RFS remained higher with GEM/DOCE at 78% compared with 62% with BCG, and at 36 months was 66% versus 60%. HG-RFS followed a similar pattern, with consistently higher rates observed in the GEM/DOCE group. Despite greater recurrence control survival, PFS, CSS, OS, and CFS remained high and comparable between treatment groups, indicating preserved oncologic safety. From a tolerability standpoint, treatment discontinuation due to intolerance occurred less frequently with GEM/DOCE, affecting 2.9% of patients compared with 9.2% in the BCG group, and grade 3–5 adverse events were also less common, occurring in 1.4% versus 4.0%, respectively.

These findings were further supported by the comparative analysis reported by Abou Chakra et al., which similarly demonstrated higher RFS and HG-RFS with GEM/DOCE across all time points. In that study, RFS at 6, 12, 24, and 36 months was consistently higher with GEM/DOCE than with BCG, while PFS, CSS, OS, and CFS remained comparable between treatment groups throughout follow-up. Although overall adverse event rates were similar, intolerance to induction therapy and treatment discontinuation were more frequent in the BCG group, whereas severe toxicity remained uncommon in both cohorts.

Collectively, these concordant findings reinforce the role of GEM/DOCE as a promising bladder-preserving intravesical option in selected patients; nonetheless, given the retrospective nature of both studies and the inherent risk of selection bias, these observations should not be interpreted as definitive evidence of superiority over BCG (13, 15).

Beyond efficacy and tolerability, the practical and economic profile of GEM/DOCE warrants consideration alongside its oncologic outcomes. From a cost perspective, a preliminary cost-effectiveness analysis suggested that GEM/DOCE may represent a less costly option compared with BCG in the BCG-naïve high-risk NMIBC setting, with mean 2-year costs of $7,090 versus $12,363 per patient at equivalent effectiveness of 1.76 quality adjusted life years (QALYs) for both strategies (20). In contrast, FDA approved alternatives for BCG-unresponsive disease carry a substantially higher economic burden; at placeholder pricing, nadofaragene firadenovec and pembrolizumab demonstrated incremental cost-effectiveness ratios (ICERs) of $263,000 and $168,000 per QALY gained, respectively, in the CIS population, both exceeding the standard willingness-to-pay threshold of $150,000 per QALY, whereas nadofaragene approached cost-effectiveness in the non-CIS population at $145,000 per QALY gained; nonetheless, these findings require validation in prospective economic studies (21). Regarding accessibility, global BCG shortages have persistently limited the availability of standard intravesical immunotherapy, while newly FDA-approved alternatives such as nadofaragene firadenovec and pembrolizumab, despite regulatory approval, have demonstrated limited real-world uptake owing to restricted availability and high treatment costs across multiple regions (21, 22). In contrast, GEM/DOCE, consisting of two widely available generic chemotherapeutic agents, is not subject to the manufacturing and supply constraints that have historically affected BCG, potentially offering a more consistently accessible treatment option across diverse healthcare settings (20, 22). From a practicality standpoint, GEM/DOCE does not require specialized delivery infrastructure and can be administered in standard outpatient urology settings; however, the sequential nature of instillations, requiring over 120 minutes per visit, may pose tolerability and logistical challenges in certain patient populations and practice settings.

Beyond these cross-treatment comparisons, variability in treatment protocols across the included studies, particularly with respect to intravesical dwell time and maintenance scheduling, may have contributed to the variability in observed oncologic outcomes. While gemcitabine dose was largely uniform at 1 g across all included studies, emerging evidence suggests that dose escalation may influence recurrence outcomes, as 2 g gemcitabine with docetaxel resulted in superior all-grade RFS compared to the standard 1 g regimen at 12 months and 24 months, though no advantage was observed for high-grade RFS and no significant difference in adverse events was noted between the two dosing regimens (23). Beyond drug dosing, the duration of intravesical dwell time also warrants consideration; in contrast to dose, prolonged dwell time administration was not significantly associated with improved recurrence or survival outcomes compared with shorter dwell time combinations, indicating that extending exposure duration beyond standard protocols may not confer additional oncologic benefit (10). Nevertheless, standard sequential dwell times pose a tolerability challenge in approximately 20% of patients due to inadequate bladder capacity or detrusor overactivity, and repeated instillations have been associated with cumulative bladder irritation and a fourfold increase in bladder-relaxing medication use, suggesting that treatment burden may cumulatively affect long-term adherence (24, 25). Of all protocol variables examined, maintenance therapy demonstrated the strongest and most consistent association with oncologic outcomes; several studies consistently demonstrated that maintenance significantly improves recurrence control across all-grade and high-grade outcomes (10, 16, 23, 26). However, tolerability challenges remain significant; BCG-based maintenance data demonstrate that the tolerability burden increases with longer treatment duration, with withdrawal rates of up to 90% reported in 3-year maintenance protocols, only 10% of patients completing the full course, and most discontinuations occurring during the induction phase due to the burden of weekly administrations (25). Furthermore, evidence from the NIMBUS trial cautions that reducing maintenance frequency, while associated with fewer adverse events, significantly increased recurrence rates without improving quality of life, reinforcing that the optimal duration and frequency of GEM/DOCE maintenance remain undefined and represent a critical gap for future prospective investigation (25).

Evaluation of these findings must be considered in the context of several important limitations. Most of the included studies were retrospective cohort analyses, which inherently introduce selection bias and limit the ability to draw causal inferences. There was marked heterogeneity across studies with respect to baseline patient characteristics, tumor stage and grade distributions, risk classification, and reporting of BCG exposure and BCG failure, thereby limiting direct comparability of outcomes. Individual cohorts variably employed terms such as BCG- unresponsive, BCG-refractory, BCG-relapsing, and BCG-intolerant disease, each of which carries distinct clinical implications and prognostic significance. Follow-up duration varied substantially, ranging from approximately 9 to 49 months, and long-term oncologic outcomes beyond five years were reported in only a single cohort, restricting evaluation of treatment durability and limiting the ability to determine whether the observed recurrence control reflects durable long-term disease suppression or early treatment response. In addition, several studies enrolled relatively small patient populations, reducing the precision of reported estimates, and overall study quality was variable, with two studies rated as poor owing to nonuniform population selection and incomplete or inconsistent reporting of GEM/DOCE treatment exposure and maintenance strategies. The inclusion of these studies may have introduced variability into the pooled outcome estimates, particularly for the BCG-failure cohort, and conclusions drawn from this subgroup should therefore be interpreted with appropriate caution. Several included studies share common investigators and originate from overlapping institutional cohorts, raising the possibility of duplicated patient populations, which may influence the interpretation of the overall evidence and should be considered when drawing conclusions from this review.

Statistical heterogeneity was substantial for several outcomes, reflecting variation in patient populations, prior BCG exposure, and treatment protocols across the included studies; pooled estimates for these outcomes should therefore be interpreted with appropriate caution. Furthermore, restriction to English publications introduces the possibility of language bias, and publication bias cannot be excluded, as studies reporting favorable outcomes are generally more likely to be published than those with negative or inconclusive results, which may lead to an overestimation of treatment efficacy and further limit the generalizability of these findings. Despite these limitations, the collected evidence supports intravesical GEM/DOCE as a viable bladder-preserving treatment option for patients with high- and very-high–risk NMIBC. The regimen appears particularly effective in BCG-naïve disease and offers a reasonable salvage strategy following BCG-failure, especially for patients seeking alternatives to early radical cystectomy or in settings of BCG shortage.

Future research should prioritize prospective, adequately powered studies with standardized definitions of NMIBC risk categories and BCG-naïve or failure, prespecified time-to-event reporting, inclusion of intermediate-risk and BCG-exposed populations with risk-stratified outcomes, and extended follow-up to more clearly define durability of response, progression risk, cystectomy rates, and survival outcomes.

## 5 Conclusion

Intravesical gemcitabine and docetaxel (GEM/DOCE) represents an effective and well-tolerated intravesical therapeutic option in patients with high- and very high-risk NMIBC, demonstrating favorable recurrence outcomes with preserved survival. The regimen appears particularly effective in BCG-naïve disease, while also providing clinically meaningful disease control in BCG-failure populations. However, the available evidence is limited by heterogeneity across studies and the predominance of retrospective designs. Further prospective, adequately powered studies are required to better define its role and optimize treatment protocols.

## Data Availability

The original contributions presented in the study are included in the article/**Supplementary Material**. Further inquiries can be directed to the corresponding authors.
