# Supplementary material for: Sequential gemcitabine–docetaxel in BCG-naïve and BCG-failure non–muscle-invasive bladder cancer: a systematic review and meta-analysis
Source: Front Oncol. 2026 Jul 9;16:1859461. doi: 10.3389/fonc.2026.1859461 (PMC13391329; doi:10.3389/fonc.2026.1859461)
Supplement: Supplementary file 1 [file Table1.docx]

**Supplementary Table 1: Risk of Bias Assessment of Included Studies**

| **Section A: Observational Cohort and Cross-Sectional Studies Tool** | | | | | | | | | | | | | | | | |
| --- | --- | --- | --- | --- | --- | --- | --- | --- | --- | --- | --- | --- | --- | --- | --- | --- |
| **Author, Year** | **Q1: Research Question** | **Q2: Study Population** | **Q3: Participation Rate ≥50%** | **Q4: Uniform Selection Criteria** | **Q5:**  **Sample Size Justification** | **Q6: Exposure Before Outcome** | **Q7: Sufficient Timeframe** | **Q8: Exposure Levels Examined** | **Q9: Exposure Measure Validity** | **Q10: Repeated Exposure** | **Q11:**  **Outcome Measure Validity** | **Q12: Outcome Assessor Blinding** | | **Q13:**  **Loss to Follow-up ≤20%** | **Q14: Confounding Controlled** | **Overall Quality Rating** |
| McElree, 2023 | Y | Y | NR | Y | N | Y | Y | NA | Y | NA | Y | NA | NR | | N | Good |
| Bakula, 2024 | Y | Y | NR | Y | N | Y | Y | NA | Y | NA | Y | NA | NR | | N | Good |
| Refugia, 2024 | Y | Y | NR | Y | N | Y | Y | NA | Y | NA | Y | NA | NR | | N | Good |
| Abou Chakra, 2025 | Y | Y | NR | Y | N | Y | Y | NA | Y | NA | Y | NA | NR | | Y | Good |
| Steinberg, 2020 | Y | N | NR | N | N | Y | Y | NA | N | NA | Y | NA | NR | | N | Poor |
| Yim, 2023 | Y | Y | NR | N | N | Y | Y | NA | Y | NA | Y | NA | NR | | N | Fair |
| Chevuru, 2023 | Y | Y | NR | Y | N | Y | Y | NA | Y | NA | Y | NA | NR | | N | Good |
| Scilipoti, 2025 | Y | Y | Y | N | N | Y | Y | NA | N | NA | Y | NA | NR | | N | Poor |
| **Section B: Before-After (Pre-Post) Studies With No Control Group Tool** | | | | | | | | | | | | | | | | |
| **Author, Year** | **Q1:**  **Study Question Stated** | **Q2: Eligibility Criteria Prespecified** | **Q3: Participants Representative** | **Q4:**  **All Eligible Enrolled** | **Q5:**  **Sample Size Sufficient** | **Q6: Intervention Clearly Described** | **Q7: Outcome Measures Valid** | **Q8: Outcome Assessors Blinded** | **Q9:**  **Loss to Follow-up ≤20%** | **Q10: Statistical Methods (Pre-Post)** | **Q11:**  **Multiple Outcome Measurements** | **Q12:**  **Group-Level Analysis** | **N/A** | | **N/A** | **Overall Quality Rating** |
| Patel, 2024 | Y | Y | Y | NR | Y | Y | Y | NA | Y | Y | NA | NA | — | | — | Good |

*Y, Yes; N, No; NR, Not Reported; NA, Not Applicable*

**Section A — Domain Descriptions (NHLBI Quality Assessment Tool for Observational Cohort and Cross-Sectional Studies):**

**Q1:** Was the research question or objective in this paper clearly stated?

**Q2:** Was the study population clearly specified and defined?

**Q3:** Was the participation rate of eligible persons at least 50%?

**Q4:** Were all the subjects selected or recruited from the same or similar populations (including the same time period)? Were inclusion and exclusion criteria prespecified and applied uniformly to all participants?

**Q5:** Was a sample size justification, power description, or variance and effect estimates provided?

**Q6:** For the analyses in this paper, were the exposure(s) of interest measured prior to the outcome(s) being measured?

**Q7:** Was the timeframe sufficient so that one could reasonably expect to see an association between exposure and outcome if it existed?

**Q8:** For exposures that can vary in amount or level, did the study examine different levels of the exposure as related to the outcome?

**Q9:** Were the exposure measures (independent variables) clearly defined, valid, reliable, and implemented consistently across all study participants?

**Q10:** Was the exposure(s) assessed more than once over time?

**Q11:** Were the outcome measures (dependent variables) clearly defined, valid, reliable, and implemented consistently across all study participants?

**Q12:** Were the outcome assessors blinded to the exposure status of participants?

**Q13:** Was loss to follow-up after baseline 20% or less?

**Q14:** Were key potential confounding variables measured and adjusted statistically for their impact on the relationship between exposure(s) and outcome(s)?

**Section B — Domain Descriptions (NHLBI Quality Assessment Tool for Before-After [Pre-Post] Studies With No Control Group):**

**Q1:** Was the study question or objective clearly stated?

**Q2:** Were eligibility/selection criteria for the study population prespecified and clearly described?

**Q3:** Were the participants in the study representative of those who would be eligible for the test/service/intervention in the general or clinical population of interest?

**Q4:** Were all eligible participants that met the prespecified entry criteria enrolled?

**Q5:** Was the sample size sufficiently large to provide confidence in the findings?

**Q6:** Was the test/service/intervention clearly described and delivered consistently across the study population?

**Q7:** Were the outcome measures prespecified, clearly defined, valid, reliable, and assessed consistently across all study participants?

**Q8:** Were the people assessing the outcomes blinded to the participants' exposures/interventions?

**Q9:** Was the loss to follow-up after baseline 20% or less? Were those lost to follow-up accounted for in the analysis?

**Q10:** Did the statistical methods examine changes in outcome measures from before to after the intervention? Were statistical tests done that provided p values for the pre-to-post changes?

**Q11:** Were outcome measures of interest taken multiple times before the intervention and multiple times after the intervention (i.e., did they use an interrupted time-series design)?

**Q12:** If the intervention was conducted at a group level did the statistical analysis take into account the use of individual-level data to determine effects at the group level?
